# Characterization and Anti-Allergic Mechanisms of Bioactive Compounds in a Traditional Chinese Medicine Prescription Using UHPLC-Q-TOF-MS/MS, Network Pharmacology and Computational Simulations

**DOI:** 10.3390/ph18101444

**Published:** 2025-09-26

**Authors:** Liang Hong, You Qin, Chiwai Ip, Wenfei Xu, Haoxuan Zeng, Xiu Duan, Ji Wang, Jing Zhao, Qi Wang, Shaoping Li

**Affiliations:** 1 Joint Laboratory of Chinese Herbal Glycoengineering and Testing Technology, University of Macau & National Glycoengineering Research Center, Macau 999078, China; yc07543@um.edu.mo (L.H.); qinyou2018@163.com (Y.Q.); mc15369@um.edu.mo (C.I.); mc25541@um.edu.mo (W.X.); mc25534@um.edu.mo (H.Z.); mc25529@um.edu.mo (X.D.); jingzhao@um.edu.mo (J.Z.); 2 State Key Laboratory of Mechanism and Quality of Chinese Medicine, Institute of Chinese Medical Sciences, University of Macau, Macau 999078, China; 3 Department of Pharmaceutical Sciences, Faculty of Health Sciences, University of Macau, Macau 999078, China; 4 National Institute of TCM constitution and Prevention Medicine, Beijing University of Chinese Medicine, Beijing 100029, China; doctorwang2009@126.com; 5 Macao Centre for Testing of Chinese Medicine, University of Macau, Macau 999078, China

**Keywords:** allergy, allergic constitution prescription, UHPLC-Q-TOF-MS/MS, network pharmacology, molecular docking, molecular dynamics simulation

## Abstract

**Background/Objectives:** Allergic diseases (e.g., asthma, chronic urticaria) are increasing globally, but current anti-allergic drugs exhibit limitations in efficacy and safety. Traditional Chinese Medicine (TCM) emphasizes constitutional regulation for allergic diseases management. The allergic constitution prescription (ACP), a TCM formulation, lacks clear mechanistic insights. **Methods:** This study employs a novel network pharmacology approach integrating ultra-high performance liquid chromatography quadrupole time-of-flight tandem mass spectrometry (UHPLC-Q-TOF-MS/MS) to identify ACP’s chemical components and compare its mechanisms with anti-allergic drugs. Chemical components of ACP were analyzed via UHPLC-Q-TOF-MS/MS, and allergic disease-related targets were collected from public databases. Anti-allergic drug targets were intersected with ACP-disease targets to identify unique and common pathways. Molecular docking and dynamics simulations assessed binding affinity between key compounds and core targets. **Results:** We identified 126 compounds in ACP. Compared to anti-allergic drugs, ACP targeted 10 unique and five common key pathways (e.g., MAPK signaling), 10 unique and nine common core targets (e.g., Tumor Necrosis Factor (TNF), IL-6), and 14 unique and 15 common key compounds. Simulations confirmed high binding affinity of ACP compounds to core targets. **Conclusions:** These findings highlight ACP’s potential multi-target mechanisms for allergic diseases treatment, identifying unique and shared pathways, targets, and compounds compared to anti-allergic drugs, offering new insights for further mechanistic studies. However, it is crucial to note that these mechanistic predictions and compound-target interactions are primarily derived from computational analyses, and experimental validation (e.g., in vitro or in vivo assays) is essential to confirm these computational findings.

## 1. Introduction

Allergic diseases, a group of conditions caused by the immune system’s hypersensitivity to typically harmless environmental substances (known as allergens), such as asthma, chronic urticaria, and pollinosis, have seen a rising incidence globally, becoming a common health concern that significantly impacts people’s lives [1]. According to the data of the World Allergy Organization (WAO), the global incidence rate of allergic reactions ranges from 50 to 112 cases per 100,000 people per year, and the estimated lifetime prevalence is 0.3% to 5.1% [2]. The World Health Organization (WHO) reports that allergic diseases now rank as the fourth most prevalent chronic condition globally, with projections indicating that half of the world’s population could be impacted by 2050 [3]. In China, over 1% of people suffer from chronic urticaria, and this number is still increasing [4]. More than 30% of Japan’s population suffers from pollinosis (pollen-induced allergic disorder) [5], and over 200 people die from allergic reactions in the United States each year [6].

To date, there are 6.5% to 35.0% of cases of idiopathic allergic reactions in which the triggering factors cannot be identified, and there is no ideal treatment for systemic allergic reactions [2]. Inadequate diagnosis and treatment remain international public health issues [7]. Currently, Western medicine mainly uses desensitization and symptomatic treatment to control allergic diseases. Epinephrine is the recommended first-line drug for the treatment of systemic allergic reactions. Second-line drugs include β2-adrenergic agonists, glucocorticoids, and antihistamines. Despite offering prompt relief for various allergic diseases, these medications do not address the root cause of allergic reactions, and the risk of potential adverse effects restricts their clinical utility [2,8]. Therefore, it is necessary to explore new effective and safe methods for treating allergic diseases.

The concept of “preventive treatment” in traditional Chinese medicine (TCM) has unique advantages in preventing of allergic diseases. Wang Qi, an academician of the Chinese academy of engineering, proposed the diagnosis and treatment model of “constitution differentiation-disease differentiation-syndrome differentiation”, constructed the theory of allergic constitution, and pointed out that the occurrence of allergic diseases is related to allergic constitution, which is the “common soil” for the occurrence of allergic diseases [9,10]. The theory of allergic constitution shifts the focus of prevention from “disease” to “person”, reflecting the concept of “people-oriented and tailored to the individual” in TCM [11]. In recent years, studies have confirmed the existence of an allergic constitution. Goksör et al. [12] pointed out that allergy process involves the coexistence of allergic disease-related patterns, rather than the gradual development of a single allergic disease. Ferreira et al. [13] showed that eczema, hay fever and asthma have common genetic risk genes. Therefore, the key to preventing and treating allergic diseases lies in regulating the “physique soil”. For allergic constitution, Chinese engineering academician Wang Qi proposed a TCM treatment prescription, i.e., allergic constitution prescription (ACP), which is composed of 13 kinds Chinese herbs, including *Ginkgo biloba* (Bai Guo, BG), *Angelica dahurica* (Bai Zhi, BZ), *Lilium brownie* (Bai He, BH), *Mentha haplocalyx* (Bo He, BoH), *Citrus reticulata* (Chen Pi, CP), *Ziziphus jujuba* (Da Zao, DZ), *Glycyrrhiza uralensis* (Gan Cao, GC), *Pueraria lobata* (Ge Gen, GG), *Agastache rugosa* (Huo Xiang, HX), *Lonicera japonica* (Jin Yin Hua, JYH), *Prunus mume* (Wu Mei, WM), *Houttuynia cordata* (Yu Xing Cao, YXC) and *Perilla frutescens* (Zi Su Ye, ZSY).

**Figure 1:**
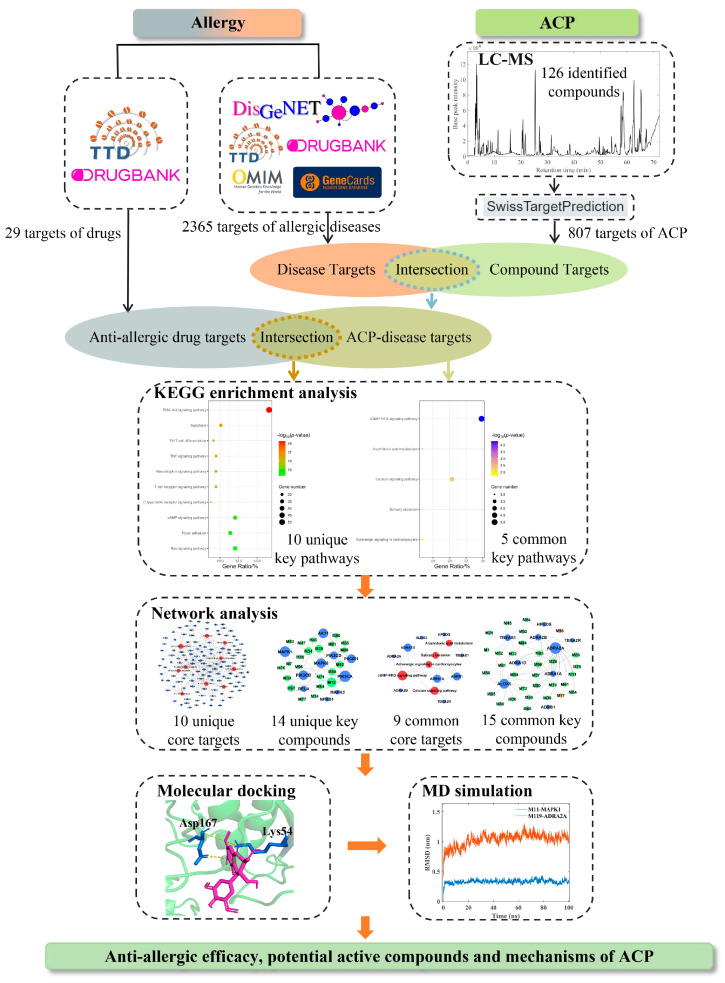
The research flowchart of this study.

The inherent complexity of TCM prescriptions, characterized by numerous chemical components and their multifaceted pharmacological effects, poses significant challenges in elucidating their precise mechanisms of action against diseases like allergic conditions. Specifically for ACP, while it holds empirical value in clinical practice for allergic constitution, its underlying molecular mechanisms, key active compounds, and target pathways have yet to be systematically and comprehensively characterized by modern scientific methods. This lack of clear mechanistic insights necessitates a robust, integrative approach. To overcome these challenges and provide a comprehensive understanding of ACP’s intricate actions, this study employs a novel and integrative approach. It combines ultra-high performance liquid chromatography quadrupole time-of-flight tandem mass spectrometry (UHPLC-Q-TOF-MS/MS) for precise chemical characterization of the complex chemical components within ACP, establishing the material basis for subsequent network pharmacology; network pharmacology based on anti-allergic drug target information for systems-level mechanistic insights into ACP’s multi-target and multi-pathway actions, thereby prioritizing potentially relevant compounds and pathways from the complex mixture; and molecular docking and molecular dynamics (MD) simulation for detailed analysis of microscopic molecular interaction patterns between key compounds and core targets, aiming to comprehensively explore the potential active compounds and mechanisms of ACP. The research flowchart is shown in Figure 1.

## 2. Results

### 2.1. Qualitative Study of Chemical Constituents in ACP Based on UHPLC–Q–TOF–MS Technology

This study utilized UHPLC-Q-TOF-MS/MS for high-sensitivity and high-resolution analysis of the complex chemical composition of ACP. Using UHPLC-Q-TOF-MS/MS analysis, a total of 126 compounds were identified or provisionally characterized in the ACP. The results of the identified compounds are shown in Table S1, while the base peak chromatograph (BPC) in positive mode is shown in Figure S1. The mass accuracy for the molecular ions of all identified compounds was maintained within a range of ±6 ppm.

Based on the results, the identified compounds were divided into seven types, including 58 flavonoids, 14 amino acids, 12 phenolic acids, 9 saponins, 7 nucleosides, 5 iridoids, and 21 other compounds based on their structural characteristics. The fragmentation patterns proposed for various compound classes are detailed below.

#### 2.1.1. Flavonoids

A total of 58 flavonoids were characterized in ACP, most of which are isoflavones or dihydroflavones. Puerarin, daidzin, genistin and other isoflavones are the characteristic compounds of *Pueraria lobata*, liquiritin and its derivatives are the unique compounds of *Glycyrrhiza uralensis*, naringin and its derivatives are the biomarkers of *Citrus reticulata*, and other compounds are common in plant medicinal materials. Previous literature has systematically discussed the mass fragment pathway of flavones, including *O*- and *C*-glycosyl flavones in accordance with different connection modes between glycosides and aglycones [14]. For O-glycosyl flavonoids, characteristic fragment ions of flavonoid glycosides, including those derived from pentacarosaccharides, hexacarosaccharides, and hexacarosaccharide derivatives with mass losses of *m/z* 132, 146, 162, and 176 Da, were detected simultaneously in the mass spectrometry data. Taking Daidzin (*m/z* 417.1192, [M + H]^+^, No. **62**) as an example, it produced a fragment ion *m/z* 255.0661 through O-cleavage (shown in Figure S2A). For *C*-glycosyl flavones, the product ions of [M + H − C_4_H_8_O_4_]^+^ or [M + H−C_5_H_10_O_5_]^+^ were observed, resulting from cleavage within the saccharide ring, and Retro−Diels−Alder fragmentation was undergone on the *C*–ring [15] (shown in Figure S2B). For the glycosides that have lost glycosyls, in addition to the general loss of H_2_O (18 Da) and CH_3_ (15 Da), the skeleton mainly generates corresponding fragment ions through RDA cleavage of the *C*-ring, and easily loses CO (28 Da), CO_2_ (44 Da), C_2_H_2_O (50 Da), and –C_3_O_2_ (68 Da), leading to the formation of corresponding characteristic ions. Similarly, other 56 flavonoids were identified according to the above mass spectrometry fragmentation rules [16].

#### 2.1.2. Amino Acids

Fourteen amino acids were analyzed in positive ion mode. Most amino acids exhibited characteristic neutral losses of NH_3_ and HCOOH [17,18], including pyroglutamic acid (No. **5**), tyrosine (No. **10**), leucine (No. **12**), and phenylalanine (No. **20**), among others. Tyrosine is presented as a representative example to illustrate their fragmentation patterns in mass spectrometry. Tyrosine showed the [M + H]^+^ ion at *m/z* 182.0812, while its fragment ions at *m/z* 165.0553 [M + H-NH_3_]^+^, *m/z* 136.0764 [M + H-HCOOH]^+^, and *m/z* 147.0445 [M + H-NH_3_-OH]^+^, were detected (Figure S3). The remaining 13 amino acids were identified by comparing their accurate masses and cleavage patterns.

#### 2.1.3. Phenolic Acids

In the MS/MS spectrum, phenolic acids, including regalosides A and D, caffeic acid, chlorogenic acid, isochlorogenic acid, 4-hydroxybenzoic acid etc., were easily identified in ACP. Take regaloside A (*m/z* 401.1442, [M + H]^+^, No. **48**) as a representative compound. It initially loses a glycosyl moiety, yielding a fragment ion at *m/z* 239.0920. Subsequent cleavage of the carboxyl and hydroxyl groups results in a further fragment ion at *m/z* 147.0447 (Figure S4) [19]. After phenolic oriented data-mining, a total of 12 phenolic acids have been discovered in present study.

#### 2.1.4. Saponins

A total of nine saponins, including licorice-saponin G_2_ and its three isomers, glycyrrhizic acid, uralsaponin A and uralsaponin B, were analyzed in the present study. Among these identified compounds, glycyrrhizic acid, one of the most abundant compounds, were considered as one of the bioactive marker compounds of Gancao or its products. Glycyrrhizic acid (No. **123**) was detected at *m/z* 823.4097 ([M + H]^+^), and produced three characteristic fragment ions at *m/z* 647.3780, *m/z* 471.3464, and *m/z* 453.3365, corresponding to [M + H − glucuronic acid]^+^, [M + H–2 × glucuronic acid]^+^ and [M + H − 2 × glucuronic acid − H_2_O]^+^, respectively (Figure S5). By analyzing the fragmentation patterns of glycyrrhizic acid and comparing them with existing literature, nine triterpene saponins were identified, exhibiting similar mass spectrometry characteristics [15,20].

#### 2.1.5. Nucleosides

Based on existing studies, the primary fragmentation pattern of nucleosides involves the loss of a glycosyl group or the cleavage of a six-membered ring structure [18]. For example, adenosine (No. **11**) easily loss of glycosyl to get fragment ion *m/z* 136.0626, meanwhile guanosine (No. **17**) and uridine (No. **14**) were observed lose glycosyl and HCOOH to achieve *m/z* 152.0578 and *m/z* 113.0351, respectively. Fragment ions *m/z* 136.0626 and *m/z* 152.0578 are hexagonal rings containing N, which are prone to ring opening and loss of NH_3_, resulting in the formation of corresponding fragment ions (Figure S6).

#### 2.1.6. Iridoids

Through the HPLC-MS of ACP, five iridoids were identified, including secologanic acid or isomer, secoxyloganin or isomers, and sweroside. These compounds predominantly undergo cleavage of glycosyl units and neutral molecules, with sweroside exemplified as a representative case to illustrate their mass spectrometry fragmentation patterns. Sweroside (No. **47**) showed the [M + H]^+^ ion at *m/z* 359.1286, and its fragment ions at *m/z* 197.0813 [M + H-Glc]^+^, *m/z* 179.0706 [M + H-Glc-H_2_O]^+^, and *m/z* 151.0764 [M + H-Glc-H_2_O-CO]^+^ were detected (Figure S7). Sweroside was characterized by its accurate mass and alignment with fragmentation patterns.

#### 2.1.7. Other Compounds

In addition to the above identified compounds, 21 other compounds, including five coumarins (dillapiol, 8-hydroxybergapten, byakangelicol, phellopterin, and apiole), two phenylpropanoids (lyoniresinol, glycyrol), cathinone, 2-formylcinnamic acid, tormentic acid, heptopyranosides, loganin, *epi*-vogeloside, limonin, 7-*O*-ethyl sweroside, pueroside B, nomilin, dillapiol, sophoraside A, sweroside aglycone and aurantiamide acetate, were identified from ACP. Overall, the mass spectrometry fragmentation of these compounds is mainly due to the loss of glycosyl or neutrality. For example, Pueroside B (*m/z* 637.2127, No. **81**), an aromatic glycoside derived from Pueraria lobata, which can be obtained fragment ions *m/z* 475.1598 [M + H–2 × glu]^+^, *m/z* 313.1077 [M + H–2 × glu]^+^ by continuously removing glucose. Their detailed mass spectrum information is shown in Table S1.

### 2.2. Target Analysis Results

**Figure 2:**
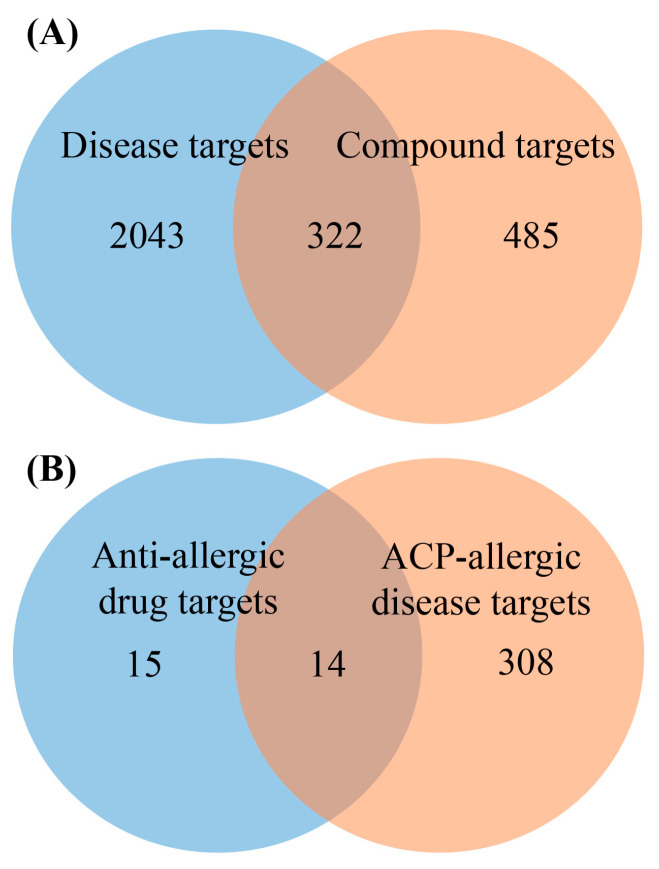
(**A**) Venny map of ACP compound targets and allergic disease targets. (**B**) Venn map of anti-allergic drug targets and ACP-allergic disease targets.

126 identified compounds were predicted by SwissTargetPrediction to obtain 807 targets. A total of 2365 disease-related targets were gathered from the Genecards, OMIM, DisGeNET, TTD, and Drugbank databases. The intersection between the targets of ACP compounds and the disease targets was analyzed, resulting in 322 shared targets identified as potential targets for ACP in treating allergic diseases, as illustrated in Figure 2A.

A total of 73 anti-allergic drugs were retrieved from the Drugbank and TTD databases. From these, 29 targets were identified, which are effective targets that have been proven to be acted upon by drugs to exert anti-allergic effects. By intersecting the 29 targets with the 322 targets of ACP-diseases, we can obtain 14 common targets of anti-allergic drugs and ACP-treated diseases, as well as 308 unique targets of ACP-treated diseases, as shown in Figure 2B.

### 2.3. Kyoto Encyclopedia of Genes and Genomes (KEGG) Pathway Enrichment Analysis Results

**Figure 3:**
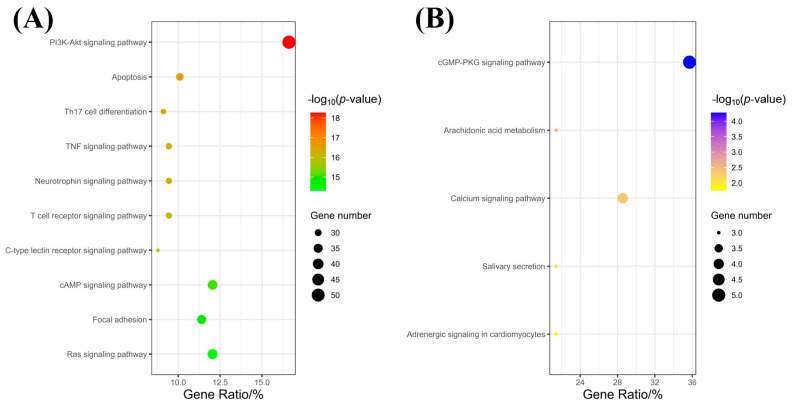
Key pathway analysis. Compared to anti-allergic drugs, ACP treatment for allergic diseases has (**A**) unique pathways and (**B**) common pathways. Color transitions from green to red (or from yellow to blue) indicate that the *p*-value decreases and the statistical significance increases.

The collected 308 unique ACP-disease targets and 14 common targets were submitted to DAVID 6.8 (https://davidbioinformatics.nih.gov/, accessed on 14 April 2025) to obtain 95 unique pathways of ACP-disease (the top 10% of pathways were taken as key pathways) and 5 common pathways (*p* < 0.05). All pathways were sorted by *p* value, and the visualization is shown in Figure 3. Figure 3A shows 10 unique key pathways of ACP-disease. Figure 3B shows 5 common key pathways. Tables S2 and S3 show a possible relationship between these potential key pathways and allergic diseases.

### 2.4. Network Analysis

**Figure 4:**
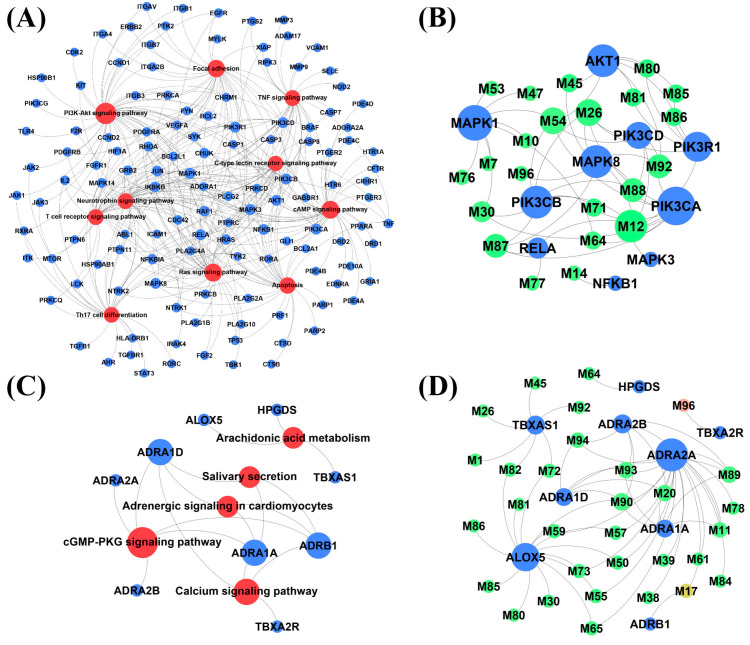
Network analysis. Compared to anti-allergic drugs, ACP treatment for allergic diseases has (**A**) a unique key pathway-target network, (**B**) a unique core target-compound network, illustrating the potential interactions between unique targets (blue nodes) and their corresponding compounds (green nodes). (**C**) a common key pathway-target network, and (**D**) a common core target-compound network, illustrating the potential interactions between common targets (blue nodes) and their corresponding compounds (green nodes). Red nodes are pathways, blue nodes are targets, and green nodes are compounds.

This study utilized 10 unique key pathways and 5 common key pathways along with their associated targets to develop key pathway-target networks, aiming to explore the core targets that underpin the similarities and differences between ACP and anti-allergic drugs in treating allergic diseases. Figure 4A illustrates that the unique network comprises 136 nodes (10 pathways, 126 targets) connected by 333 edges. Within this network, the top 10% of targets (10/126) with the highest degree values (degree > 7) were identified as core targets and are presented in Table 1. These represent potential core targets associated with ACP’s unique mechanisms in treating allergic diseases, differentiating it from conventional anti-allergic drugs. Figure 4C illustrates that the common network comprises 14 nodes (5 pathways and 9 targets) connected by 18 edges. Within this network, nine targets with a degree value of 1 or higher are listed in Table 1. These are potential core targets that reflect common mechanisms shared by ACP and anti-allergic drugs in treating allergic diseases.

The core target-compound networks were subsequently constructed based on these 10 unique and 9 common core targets and their corresponding compounds, as shown in Figure 4B,D. These networks are designed to visualize and screen for key active compounds within ACP that may interact with these core targets through direct or indirect mechanisms, thereby elucidating their unique or common anti-allergic effects. The unique core target-compound network (Figure 4B) comprises 32 nodes (10 targets, 22 compounds) connected by 54 edges. Table 2 presents 14 key compounds with a degree value exceeding 1 in the network, representing potential key compounds involved in ACP’s unique mechanism in treating allergic diseases compared to anti-allergic drugs. The common core target-compound network (Figure 4D) includes 40 nodes (9 targets, 31 compounds) connected by 57 edges. Table 2 presents 15 key compounds with a degree value of 2 or higher in this network, representing potential key compounds contributing to the common mechanisms of ACP in the treatment of allergic diseases compared to anti-allergic drugs.

### 2.5. Molecular Docking Analysis

**Figure 5:**
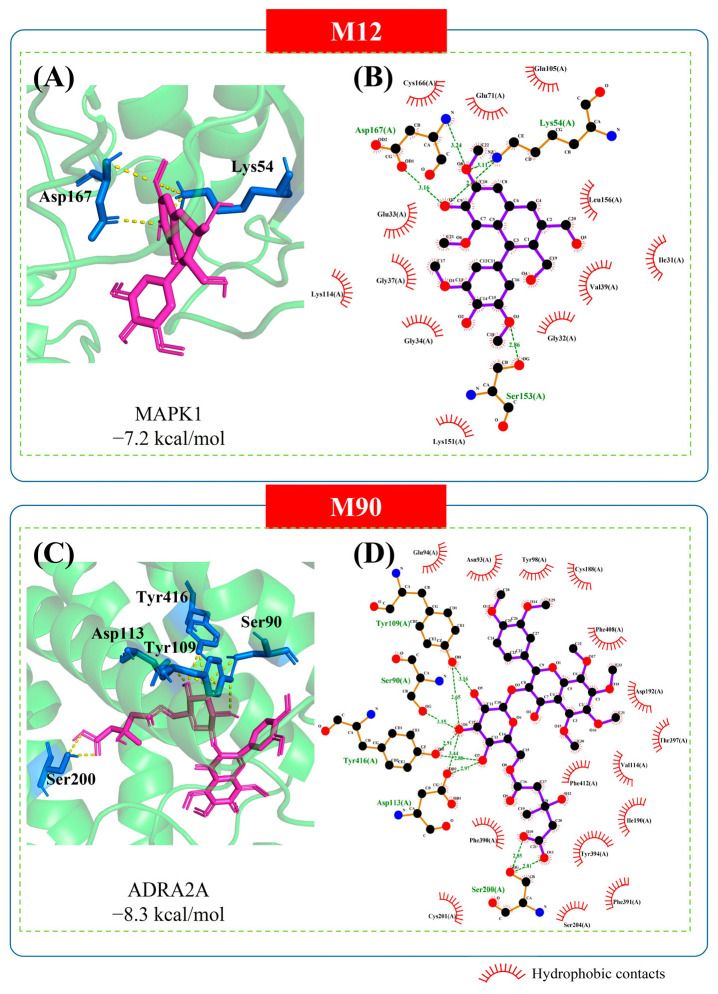
Prediction binding patterns of M12 and M90 with their respective highest-scoring targets. (**A**,**C**) were visualized using Pymol. The protein target is shown in green, the amino acid residue in marine, and the compound in light magenta. (**B**,**D**) were visualized using LigPlus+.

Molecular docking was employed to further validate the interactions between core targets and key compounds. The docking information of unique/common core targets is shown in Tables S4 and S5. The unique/common key compounds were docked with the core targets of the corresponding parts, and the docking results are shown in Tables S6 and S7, respectively. Generally, a docking score ≤ 0 kcal/mol indicates spontaneous interaction; values ≤ −7.0 kcal/mol suggest strong binding affinity, and lower (more negative) scores are associated with stronger binding activity [21]. These empirical thresholds are widely used in computational drug discovery to preliminarily assess the binding potential between ligands and targets and guide the screening of active compounds. It can be seen from the table that the docking scores of M92, M30, M45 and M11 with the key targets are all greater than −7, which indicates that they are less likely to be anti-allergic compounds than other compounds. The compounds with the highest degree values from both unique and common sections were selected for further analysis, namely M12 and M90, respectively. The degree values of the compounds are in Table 2. Their predicted binding modes with their highest scoring targets are shown in Figure 5. Among them, M12 established hydrogen bonds with Lys54 amino acid and Asp167 amino acid of MAPK1 (Figure 5A,B). M90 established hydrogen bonds with Ser90 amino acid, Tyr109 amino acid, Asp113 amino acid, Ser200 amino acid and Tyr416 amino acid of ADRA2A (Figure 5C,D). The rest showed hydrophobic interactions. Although these results suggest that the key compounds and core targets may form stable interactions, further experimental validation is needed to confirm these findings. Additionally, the precise role of these amino acids in the activity of the respective targets remains to be determined and warrants further investigation.

### 2.6. MD Simulation

**Figure 6:**
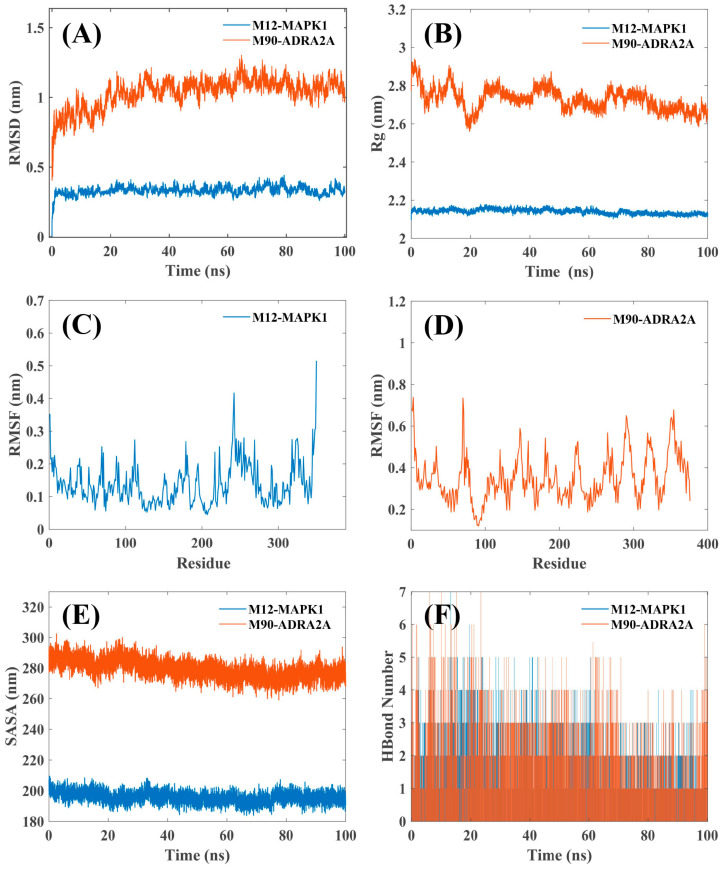
The MD simulation of the M12-MAPK1 complex and M90-ADRA2A complex. (**A**) The RMSD plot of the M12-MAPK1 complex and M90-ADRA2A complex. (**B**) The Rg plot of the M12-MAPK1 complex and M90-ADRA2A complex. (**C**) The RMSF plot of the M12-MAPK1 complex. (**D**) The RMSF plot of the M90-ADRA2A complex. (**E**) The SASA plot of the M12-MAPK1 complex and M90-ADRA2A complex. (**F**) The number of hydrogen bonds in the M12-MAPK1 complex and M90-ADRA2A complex. Blue represents the simulation results for the M12-MAPK1 complex; red represents the simulation results for the M90-ADRA2A complex.

Molecular docking predicts the static binding modes and affinities between small molecules and target proteins. To further evaluate the stability and dynamic behavior of these predicted binding modes, we performed MD simulations. MD simulations track atomic movements over a simulated timescale, offering deeper dynamic insights into the conformational changes and interactions of ligand-receptor complexes, thereby complementing and validating molecular docking results. As shown in Figure 6, we conducted MD simulations to investigate the interactions within the M12-MAPK1 and M90-ADRA2A complexes. Key parameters, including root mean squared deviation (RMSD), radius of gyration (Rg), root mean squared fluctuations (RMSF), solvent accessible surface area (SASA), and hydrogen bond interactions, were evaluated by processing and integrating the simulation outcomes.

This study utilized RMSD to analyze the mobility of the receptor-ligand complex. RMSD values quantify the deviation of atomic positions relative to the initial conformation during the simulation. Lower and stable RMSD values typically indicate a stable complex structure. Figure 6A shows the RMSD of the M12-MAPK1 and M90-ADRA2A complex. The RMSD curve shows that the M12-MAPK1 complex exhibits stable fluctuations in the time range of 0–100 ns, while the RMSD curve of the M90-ADRA2A complex tends to be stable after 20 ns.

The Rg indicates the compactness and structural constraints of the system, reflecting the extent of protein folding [22]. A higher Rg value suggests a more expanded or less compact structure of the system, which could be related to increased flexibility of the protein or ligand. Thus, a higher Rg value indicates lower stability. In contrast, a smaller Rg value suggests a compact and closely packed molecular system. As shown in Figure 6B, the Rg of the M12-MAPK1 complex remained consistent throughout the 0–100 ns simulation period, and the Rg value of the M90-ADRA2A complex exhibits a downward trend, achieving stability after approximately 80 ns.

The RMSF can indicate the complex’s fluctuation at the residue level [23]. Higher RMSF values generally suggest greater flexibility in that region. The results in Figure 6C,D show that the RMSF values of the two protein complexes are relatively low (mostly between 0.1 and 0.6 nm). However, compared with M12-MAPK1, the higher RMSF values observed in the M90-ADRA2A complex suggest greater fluctuations at the residue level, which may indicate higher flexibility in this complex. This higher flexibility may mean that ADRA2A and its complex domains have greater adaptability when interacting with different molecules, potentially allowing for induced fit mechanisms or accommodating a wider range of ligands, which could be exploited in drug design.

SASA measures the surface area of a protein exposed to solvent, reflecting changes in protein conformation and the tightness of binding. It is widely recognized that smaller fluctuations in the SASA curve indicate a more stable and compact interaction between the active compound and its target. The smaller the SASA value, the more compact the protein structure [24]. The SASA plot (Figure 6E) shows that the curves of both the M12-MAPK1 and M90-ADRA2A complex are relatively consistent and smooth. This indicates that the protein and small molecule in the two complexes are tightly bound. The larger SASA value of the M90-ADRA2A complex may suggest that some regions of the protein are more exposed or less compact, potentially indicating greater flexibility in those areas. Combining the results of RMSD, Rg, and RMSF, it can be seen that the M90-ADRA2A complex has higher dynamics and flexibility. This differential flexibility between ADRA2A and MAPK1 could imply distinct pharmacological behaviors: a more rigid target (MAPK1) might exhibit highly specific binding, while a more flexible target (ADRA2A) might allow for more diverse ligand interactions or allosteric modulation, influencing drug efficacy and selectivity.

Hydrogen bonds are relatively strong non-covalent interactions that are crucial for the stability of protein structures and ligand-receptor interactions [25]. Tracking the number of hydrogen bonds formed during the simulation provides further insight into the dynamic binding stability between the ligand and target. The number of hydrogen bonds in the M12-MAPK1 and M90-ADRA2A complexes fluctuated between 0 and 7 during the simulation time of 0–100 ns (Figure 6F). These hydrogen bonds help maintain the stability of the complexes.

## 3. Discussion

Allergic diseases, characterized by immune hypersensitivity, pose significant challenges due to their increasing prevalence and complex mechanisms. Currently, Western medicine offers diverse strategies for managing allergic diseases. While many conventional pharmacological interventions (e.g., antihistamines, corticosteroids) effectively alleviate symptoms and acute episodes, they often primarily focus on rapid symptomatic relief. This approach, though highly effective for immediate reactions, may not always address the underlying immunological dysregulation, potentially leading to recurrence upon discontinuation and concerns regarding long-term efficacy and side effects. However, it is important to acknowledge that Western medicine also includes advanced approaches like allergen-specific immunotherapy (desensitization therapy), which aims for a more fundamental immune system modulation, seeking long-term remission or a ‘cure’ by inducing immune tolerance [26]. In contrast, TCM emphasizes a holistic approach rooted in constitutional regulation and multi-level, multi-target effects, aiming to address the fundamental predisposition to allergic diseases. TCM’s philosophy also stresses treating both symptoms and root causes with typically milder effects on internal organs and fewer long-term side effects. This study explores how ACP, a TCM formulation, may exert its effects through mechanisms that complement or differ from conventional symptomatic Western treatments, potentially offering novel strategies for allergic disease management [27].

An allergic constitution, as described in the Introduction, refers to a predisposition to allergic reactions and allergic diseases, representing the “common soil” for such conditions as proposed by academician Wang Qi [9,10]. Consistent with this, people with allergic diseases can trace their family history of allergies related to bloodlines, which is a consensus in both modern Chinese and Western medicine [28]. Chinese engineering academician Wang Qi has proposed several clinical formulas to correct allergic constitutions, such as Guominkang [29], Tuo-Min-Ding-Chuan decoction [30], and Tuomin Zhiti decoction [31]. Among these, the research on Guominkang has been ongoing for years and has achieved satisfactory results [32,33,34], which demonstrates the validity and advancement of the theory of allergic constitution. Additionally, based on the theory of allergic constitution, Chinese engineering academician Wang Qi has also proposed ACP. Although there are currently no pharmacological studies on ACP, some of its individual herbals have already been investigated for their anti-allergic effects. For example, the extract of WM has the potential to inhibit mast cell degranulation [35]; the extract of YXC significantly improved airway hyperresponsiveness and inflammation in asthmatic mice [36]; ZSY is a potentially promising agent for the treatment of allergic diseases [37]. This article aims to forecast the potential active compounds and mechanisms of ACP in the treatment of allergic diseases by employing UHPLC-Q-TOF-MS/MS, network pharmacology grounded, molecular docking, and MD simulation.

In this article, an UHPLC-Q-TOF-MS method was established and performed to identify the compounds of ACP due to the few reports on their chemical compounds. A total of 126 compounds, including flavonoids, phenolic acids, phenylpropanoids, triterpenes, coumarins, saponins, iridoids, etc., were identified. The identification of such a large number of compounds highlights the inherent chemical complexity of this traditional formulation. Indeed, high-resolution mass spectrometry has been used to characterize the chemical composition of complex system with higher accuracy, sensitivity, rapidity, and comprehensiveness [38]. This advanced chemical characterization technique provides a solid foundation—the essential material basis—for the subsequent network pharmacology analysis, which is crucial for systematically discerning the potentially active components from this intricate mixture and linking them to specific anti-allergic mechanisms. While this study focuses on qualitative identification as a prerequisite for mechanistic prediction, quantitative analysis of active ingredients is certainly worth exploring in the future. Therefore, future studies will involve acquiring reference standards to validate the qualitative analysis results. Additionally, we plan to implement advanced quantitative analytical approaches, including strategies like quantitative analysis of multi-components by single-marker (QAMS) [39], digital reference standard (DRS) [40], two reference substances for determination of multiple components (TRSDMC) [41], and quantitative analysis by standardized reference extract (QASRE) [42], to systematically quantify the chemical constituents of ACP. In future studies, advanced analytical techniques, such as infrared spectroscopy and nuclear magnetic resonance, can be employed to further characterize and quantify the chemical constituents of ACP [16].

Network pharmacology, as a systematic and data-driven approach, is a modern ‘hard science’ tool critical for studying complex interactions among drugs, targets, and diseases at a network level. It is particularly adept at elucidating the multi-compound, multi-target, and multi-pathway mechanisms of complex systems like TCM, aligning profoundly with TCM’s holistic concept and syndrome differentiation philosophy. This method provides an effective and rigorous tool for the computational prediction and prioritization of active compounds and mechanisms in TCM [43,44]. The integrative strategy employed in this study, by systematically combining chemical characterization, network-level analysis, and atomic-level simulations, offers unprecedented depth and a holistic perspective into the intricate actions of complex herbal prescriptions like ACP. This advanced computational pipeline moves beyond traditional single-target drug discovery paradigms, enabling the efficient identification of both unique and common therapeutic strategies, and rigorously highlighting the inherent multi-compound, multi-target nature of TCM. Through this ‘hard science’ approach, by leveraging known anti-allergic drug target information, we were able to computationally derive the unique and common compounds, targets, and pathways of ACP therapy for allergic diseases, thereby providing a robust framework to analyze the similarities and differences in the potential compounds and mechanisms of ACP compared to anti-allergic drugs.

KEGG pathway analysis shows that the potential unique pathways of ACP treatment for allergic diseases include PI3K-Akt signaling pathway, Th17 cell differentiation, TNF signaling pathway, T cell receptor signaling pathway, etc. [45,46,47,48,49,50,51,52,53,54]. For example, the PI3K-Akt signaling pathway regulates the proliferation of airway smooth muscle cells in asthma, which is a potential pathway for treating asthma [45]. Th17 cells are one of the main cell lineages that cause allergic diseases, and the Treg/Th17 ratio is associated with allergic diseases [46]. TNF-α is associated with the occurrence of asthma [47]. Defects/changes in T cell receptor (TCR) signaling pathway transduction are associated with allergic diseases [48]. The common pathways for ACP treatment of allergic diseases and anti-allergic drugs include cGMP-PKG signaling pathway, Arachidonic acid metabolism, Calcium signaling pathway, Adrenergic signaling in cardiomyocytes, etc. [55,56,57,58,59]. The cGMP-PKG signaling pathway can regulate the contraction and relaxation of smooth muscle in airway wall vessels [55]. Arachidonic acid metabolism plays an important role in allergic reactions [56]. The calcium signaling pathway represents a potential therapeutic target for allergic asthma by regulating Th1, Th2, and Th17 cell functions [57]. Adrenergic signaling in cardiomyocytes is the key pathway for treating allergic diseases [58]. As shown above, the pathways through which ACP exerts unique anti-allergic effects are primarily associated with immune and inflammatory pathways. In contrast, the pathways through which ACP shares similarities with conventional anti-allergic drugs, such as the cGMP-PKG signaling pathway and Adrenergic signaling in cardiomyocytes, are aimed at alleviating allergic symptoms, and common drugs include Epinephrine and Xylometazoline, among others [2,60]. Meanwhile, Arachidonic acid metabolism plays an anti-inflammatory role, and common drugs for this pathway include Montelukast and Zileuton, among others [8]. More information about pathway can be seen in Tables S2 and S3.

Based on key KEGG pathway information, 10 potential core targets were obtained through the ACP unique partial key pathway target network. There have been many studies on the relationship between core targets and allergic diseases. MAPK can be a target for the development of anti-allergic drugs, and its targeted inhibitors have been validated in animal models of asthma [61]. The phosphoinositide 3-kinase (PI3K) family plays a critical role in modulating various cellular processes within the immune system, making its members promising therapeutic targets for managing allergic conditions [62]. AKT is closely related to the occurrence of allergic inflammation [63]. These findings underscore the significance of these targets in the treatment of allergic diseases and suggest that ACP may exert its therapeutic effects through multiple molecular mechanisms. In addition, the common portion of ACP and anti-allergic drugs has obtained 9 potential core targets. These serve as targets for anti-allergic drugs. They are closely related to allergic diseases. For instance, activation of α-adrenoceptor may improve symptoms associated with allergic rhinitis [64].

14 unique and 15 common potential key compounds were obtained through the core target-compound network, respectively. Among these compounds, several exhibit specific anti-allergic properties. Lyoniresinol can inhibit the IgE-mediated degranulation of mast cells, thereby preventing allergic reactions [35]. Limonin suppresses the production of IgE in human B cells and peripheral blood mononuclear cells derived from individuals with food allergies [65]. Phellopterin has been proven to be effective in treating atopic dermatitis [66]. Byakangelicol dose-dependently reduces IL-1β-induced COX-2 expression and prostaglandin E2 release [67]. Caffeine acid can inhibit various itch transmission pathways in mice [68]. Naringen can inhibit degranulation and alleviate allergic symptoms by inhibiting AKT phosphorylation [69]. Kaempferol and quercetin can effectively inhibit the development of IgE-mediated allergic inflammation in intestinal cell models [70]. Glycyrhizic acid can regulate the IgE-mediated allergic response of allergy related immune cells [71]. Other compounds, including luteolin [72], rutin [73] and daidzein [74] have been proven to have anti-allergic effects. Furthermore, nomilin [75,76], dillapiole [77,78], licorice-saponin G_2_ [79], diosmin [80], hyperside [81], and genistein [82] all have anti-inflammatory effects. As can be seen from the above information, a variety of ACP compounds act on IgE-related pathways, including Lyoniresinol, Limonin, etc. Some compounds also have specific anti-inflammatory effects, including nomilin, dillapiol, etc. Existing evidence shows that current anti-allergy drugs mainly act on specific targets like histamine receptors and leukotriene receptors, working by inhibiting histamine release, antagonizing histamine receptors, and suppressing inflammation [8]. Therefore, the main potential mechanism shared by ACP compounds and anti-allergic drugs is their anti-inflammatory effects. However, due to limited research, the pharmacological activities of ACP compounds also need to be further explored in the future.

In addition, molecular docking and dynamics simulations were employed to further validate the binding affinities and stability of these key compounds with their core targets, and to contextualize their known pharmacological effects and functional implications in clinical allergic responses. Specifically, M12 (Lyoniresinol), identified as a unique key compound and known to inhibit IgE-mediated mast cell degranulation, demonstrated strong binding affinity to MAPK1, a crucial target in asthma [61]. This interaction suggests M12′s potential to modulate immune and inflammatory pathways, thereby contributing to ACP’s unique anti-allergic mechanisms, possibly by preventing early allergic responses. Similarly, M90 (Naringin-3-O-(3-hydroxy-3-methylglutarate)-glucoside), a key compound in the common network, exhibited stable binding with ADRA2A. Activation of α-adrenoceptors like ADRA2A is known to alleviate allergic symptoms, such as those in bronchial asthma [83], highlighting M90′s role in common anti-allergic pathways shared with conventional drugs. The detailed binding modes (Figure 5), showing multiple hydrogen bonds and hydrophobic interactions, along with favorable docking scores for most key compounds, support the functional implications of these specific compound-target interactions. For instance, M12 formed hydrogen bonds with Lys54 and Asp167 of MAPK1, while M90 established multiple hydrogen bonds with Ser90, Tyr109, Asp113, Ser200, and Tyr416 of ADRA2A. The subsequent MD simulations (Figure 6) further confirmed relatively stable fluctuations for both M12-MAPK1 and M90-ADRA2A complexes throughout the 100 ns simulation (RMSD, Rg, RMSF, and SASA plots, Figure 6). Although the M90-ADRA2A complex exhibited higher flexibility in some regions (RMSF and SASA plots), which could allow for diverse ligand interactions or allosteric modulation, both complexes maintained stable hydrogen bond formation (Figure 6F). This differential flexibility between MAPK1 (more rigid) and ADRA2A (more flexible) suggests distinct pharmacological behaviors, collectively contributing to ACP’s potential multi-target, combinatorial effects in addressing various facets of allergic diseases, from immune modulation to symptom alleviation.

**Figure 7:**
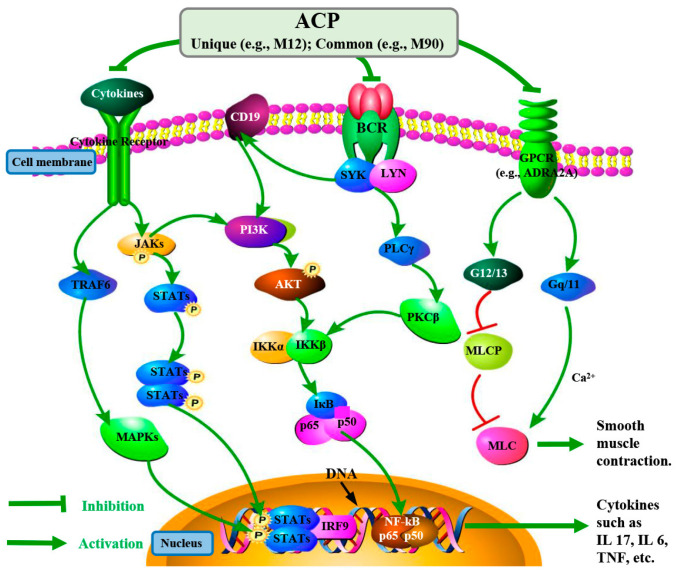
The potential mechanism of ACP in the treatment of allergic diseases.

The anti-allergic mechanisms and active compounds of ACP were elucidated through KEGG pathway and network pharmacology analyses. Figure 7 visually encapsulates several potential mechanisms by which ACP exerts its anti-allergic effects, highlighting both its unique immunomodulatory and common symptom-alleviating pathways. On the unique immunomodulatory front, ACP appears to intervene in key signaling cascades critical for allergic inflammation. For instance, ACP inhibits cytokine receptor signaling, which is initiated at the cell membrane and typically activates Tumor Necrosis Factor Receptor Associated Factor 6 (TRAF6) and further activates MAPKs [84]. Additionally, this pathway also activates Janus Kinases (JAKs) and Signal Transducers and Activators of Transcription (STATs), which then translocate to the nucleus to bind to specific DNA sequences, leading to the production of pro-inflammatory cytokines such as IL-17, IL-6, and TNF [85]. Furthermore, ACP suppresses B cell receptor (BCR) signaling, initiated at the cell membrane and involving Spleen Tyrosine Kinase (SYK) and Lck/Yes-related Novel protein tyrosine kinase (LYN), as well as the CD19-mediated Phosphoinositide 3-kinase (PI3K)-Protein Kinase B (AKT) signaling pathway [86,87]. These inhibitions converge to reduce the activation of downstream factors such as MAPKs and Nuclear Factor kappa-light-chain-enhancer of activated B cells (NF-κB), preventing their translocation to the nucleus and subsequent binding to DNA to modulate gene expression, thereby attenuating the overall inflammatory response [88]. Key compounds such as M12 are predicted to contribute to these unique immunomodulatory actions by potentially targeting core proteins like MAPK1. Concurrently, ACP also modulates pathways common to conventional anti-allergic approaches, focusing on symptomatic relief. It acts by inhibiting the activation of G Protein-Coupled Receptors (GPCRs) located on the cell membrane. Upon activation, GPCRs mediate signal transduction through G proteins (e.g., G12/13 and Gq/11) [89]. Specifically, the Gq/11 pathway typically leads to increased intracellular calcium (Ca^2+^) mobilization, often involving ion channels embedded in the cell membrane, while the G12/13 pathway inhibits Myosin Light Chain Phosphatase (MLCP), both ultimately promoting Myosin Light Chain (MLC) phosphorylation and inducing smooth muscle contraction (e.g., bronchoconstriction) [90,91]. Compounds like M90 are implicated in these common mechanisms by potentially interacting with adrenergic receptors, a type of GPCR (e.g., ADRA2A). These diverse actions underscore ACP’s multi-targeted strategy in managing allergic diseases.

Network pharmacology has been widely applied in the research of TCM [92,93], and this study uses it to predict the active compounds and mechanisms of ACP against allergic diseases. These findings provide novel strategies for developing new, safer, and more effective anti-allergic therapies. By elucidating ACP’s unique multi-target mechanisms, this study offers a scientific basis for precise treatment and personalized interventions of allergic diseases using TCM, which is expected to address the limitations of current Western medicines in efficacy and safety and potentially reduce the recurrence of allergic symptoms. However, the findings of this study are primarily derived from computational analyses, which represents a key limitation. Specifically, while molecular docking and molecular dynamics simulations offer valuable insights into potential binding interactions, and preliminarily predict compound activity through docking scores, they rely on approximations (e.g., force fields, simplified solvent models) and cannot fully capture the complexity of biological systems. Therefore, the predicted binding affinities and dynamic behaviors serve as hypotheses that require rigorous experimental corroboration to confirm their physiological relevance and therapeutic potential. Future research should focus on systematically validating the identified key compounds, core targets, and their unique and common anti-allergic mechanisms of ACP through in vitro and in vivo pharmacological experiments. Given the multi-component nature of TCM prescriptions, it is also crucial to further explore the combinatorial effects among these active compounds to fully elucidate ACP’s pharmacological actions and achieve more precise clinical applications.

## 4. Materials and Methods

### 4.1. Materials and Reagents

The BG, BZ, BH, BoH, CP, DZ, GC, GG, HX, JYH, WM, YXC and ZSY were purchased from Kangmei Pharmaceutical Co., Ltd. (Puning, Guangdong, China). HPLC-grade acetonitrile was acquired from Fisher Scientific (Geel, Belgium), and formic acid (HPLC grade, ≥99%) was obtained from Rhawn (Shanghai, China). Deionized water (18.2 MΩ/cm) was produced using a Milli-Q^®^ water purification system (Model IQ7005, Merck, Dorset, UK). Filter membranes (0.22 μm) were supplied by Cinjinghua Co. (Shanghai, China). All other reagents were of analytical grade.

### 4.2. Preparation for Water-Extraction of ACP

ACP is a TCM formulation based on the diagnostic and therapeutic model of “constitution differentiation-disease differentiation-syndrome differentiation” for allergic constitution. The specific proportions of the herbal components are derived from established TCM theory and extensive empirical clinical practice, aiming to achieve holistic regulation of the allergic constitution. To prepare the ACP, the thirteen herbal components—BG, BZ, BH, BoH, CP, DZ, GC, GG, HX, JYH, WM, YXC, and ZSY—were combined in specific ratios (9:6:10:6:6:10:9:10:10:10:10:10:10, respectively). This mixture was then transferred to a round-bottom flask, where it was steeped in 15 volumes of distilled water for 2 h, followed by a 2 h reflux extraction in a water bath. After cooling to room temperature, the resulting supernatant was filtered through a Buchner funnel. This extraction and filtration cycle was performed twice more. Finally, the collected aqueous filtrates were pooled and concentrated to a final volume of 250 mL. 50 mL of filtrate was measured out, and mixed with 200 mL ethanol, then stored overnight in a refrigerator at 4 °C. Following collection, the supernatant was filtered through a 0.22 μm PTFE membrane in preparation for UHPLC-Q-TOF-MS/MS analysis. An 80% ethanol solution served as the blank.

### 4.3. Chromatography Conditions and MS Parameters

The chromatographic separation was conducted using a Dionex Ultimate 3000 UHPLC system (Germering, Germany), which included an Ultimate 3000 degasser, pump, RS autosampler, and RS column compartment, all integrated with a diode-array detector. The separation was performed on an Agilent ZORBAX Eclipse Rapid Resolution (4.6 × 150 mm, 3.5 μm) at 30 °C. Water containing 0.1% formic acid (A) and acetonitrile (B) were used as the mobile phases. The flow rate was 0.5 mL/min, and in gradient elution: 0–10 min, 2%~10% B; 10–25 min, 10%~15% B; 25–43 min, 15–20% B; 43–53 min, 20%~40% B; 53–68 min, 40%~80% B; 68–73 min, 80% B. The sample injection volume was 2 μL. Chromatographic detection was performed at 210 nm, 254 nm, 280 nm, and 320 nm, and full UV spectra were recorded in the 190 to 400 nm range.

Mass spectrometry analysis was conducted using a high-resolution impact HD QTOF mass spectrometer (Bruker Daltonik GmbH, Bremen, Germany). Positive electrospray ionization (ESI) mode was employed for detection, with the full scan mass range set from *m*/*z* 50 to 1000. Specific MS parameters included: End Plate Offset: 500 V; Capillary: 4500 V; Nebulizer: 1.2 Bar; Dry Gas: 10.0 L/min; Dry Temp: 250 °C; Funnel 1 RF: 250.0 Vpp; Funnel 2 RF: 150.0 Vpp; isCID Energy: 0 eV; HexapoleRF: 50.0 Vpp; Ion Energy: 3.0 eV; Low Mass: 50.00 *m*/*z*; Collision Energy: 5.0 eV; Collision RF: 500.0 Vpp; Transfer Time: 65.0 μs; Pre Pulse Storage: 3.0 μs.

### 4.4. Network Pharmacology Analysis

#### 4.4.1. Collection of ACP Compound Targets and ACP-Disease Targets

Macromolecular targets for the compounds identified by UHPLC-Q-TOF-MS/MS were predicted using SwissTargetPrediction, which leverages 2D and 3D structural similarities of small molecules [94].

The Genecards database (providing gene-disease association information, version 5.11, https://www.genecards.org/, accessed on 10 April 2025), OMIM database (cataloging genetic disease information, https://omim.org/, accessed on 10 April 2025), DisGeNET database (for disease-gene association data mining, version v7.0, https://www.disgenet.org/, accessed on 10 April 2025), TTD database (offering therapeutic target data, http://db.idrblab.net/web/, accessed on 10 April 2025) and Drugbank database (containing drug and their target information, version 5.1.9, https://go.drugbank.com/, accessed on 10 April 2025) were searched using the keywords “Anaphylaxis” and “Allergy” to obtain disease targets related to allergic diseases. Subsequently, potential therapeutic targets for ACP in allergic diseases, which we termed ACP-disease targets, were identified by intersecting the compound targets with the disease targets.

#### 4.4.2. Collection of Anti-Allergic Drugs and Their Targets

Anti-allergic drugs and their targets were collected from the Drugbank and TTD database. To ensure these drugs had reached at least the clinical development stage, their status was verified against information from the U.S. Food and Drug Administration (FDA), the National Medical Products Administration (NMPA), the Pharmaceuticals and Medical Devices Agency (PMDA), and the European Medicines Agency (EMA).

#### 4.4.3. Target Intersection

By intersecting the anti-allergic drug targets with the ACP-disease targets, the unique targets of ACP for treating diseases, as well as the common targets of ACP and anti-allergic drugs for treating diseases can be obtained.

#### 4.4.4. KEGG Pathway Enrichment Analysis

To uncover the biological pathways and functional enrichments involved in ACP’s treatment of allergic diseases, the collected gene symbols of the unique targets and the common targets were imported into DAVID 6.8 (https://davidbioinformatics.nih.gov/, accessed on 14 April 2025) [95], respectively, with the Identifier, Background, and Pathways set to OFFICIAL_GENE_SYMBOL, Homo sapiens, and KEGG_PATHWAY. This process generated KEGG pathway enrichment analysis [96,97,98] results unique to ACP therapy for allergic diseases compared to anti-allergic drug therapy, as well as those common between the two.

#### 4.4.5. Network Construction

The unique and common KEGG pathway enrichment analysis results (compared to those of anti-allergic drugs) for ACP therapy in allergic diseases, along with their corresponding targets, were imported into Gephi software (version 0.9.7) to construct the unique/common key pathway-target networks. Subsequently, based on the core targets identified from the unique/common key pathway-target networks (defined as targets with higher degree values), and the ACP compounds identified by UHPLC-Q-TOF-MS/MS and predicted by SwissTargetPrediction to interact with these core targets, unique/common core target-compound networks were further constructed. These networks are designed to visualize and identify key compounds critical for ACP’s anti-allergic effects by illustrating the direct or indirect interactions between core targets and their potential active compounds. Through network analysis, the unique core targets and key compounds of ACP in the treatment of allergic diseases compared with anti-allergic drugs were identified, as well as the common core targets and key compounds.

### 4.5. Molecular Docking

To predict the binding modes and affinities between key compounds in ACP and core targets, molecular docking was performed. Three-dimensional structural data for the identified compounds were obtained from the PubChem database. For those compounds lacking existing structural information in public databases, their structures were manually drawn using ChemDraw (version 16.0, Cambridge Soft, Cambridge, MA, USA), followed by batch conversion of the SDF format files to mol2 format using Open Babel GUI (Version 2.4.1). The 3D structures of the target proteins were retrieved from the Protein Data Bank (PDB) database (http://www.rcsb.org/, accessed on 30 April 2025). For target proteins without structural information in the PDB database, the Alphafold program in uniprot (https://www.uniprot.org/, accessed on 30 April 2025) was used for prediction. Autodock Vina was used for molecular docking. The scoring function of Autodock Vina employs a hybrid empirical force field to estimate binding affinities (kcal/mol), where more negative values indicate stronger predicted binding interactions [99]. Before docking, target proteins underwent dehydration and were saved in the pdbqt format. The docking box was positioned at the co-crystallized ligand’s location within the target. For targets where no co-crystallized ligand was present, the docking box was configured to encompass the target protein as extensively as possible. The configuration file’s parameters were set as follows: exhaustiveness = 8, energy range = 3, and num modes = 9. All docking poses were ranked by their predicted binding affinity, and the highest-scoring pose for each ligand-target pair was selected for further analysis. The interaction between receptor and ligand was visualized and hydrogen bonds were displayed by Pymol (3D, version 2.4.0) and LigPlus+ (2D, version v.2.2).

### 4.6. Molecular Dynamics (MD) Simulation

To further evaluate the stability of molecular docking results and the dynamic behavior of key compound-target complexes, MD simulation was performed. MD simulation, a computational method used to simulate the physical movements of atoms and molecules over time, was performed using GROMACS (version 2018.8) [100]. For the MD simulations, the ligand topology file was generated using the Sob-top program, employing the AMBER force field, which is a widely recognized set of force fields for biomolecular simulations [101]. Concurrently, the AMBER99 force field was utilized for creating the protein topology file [102]. A dodecahedral unit cell was constructed and solvated with transferable intermolecular potential (TIP3) water molecules. Prior to the MD run, each system was neutralized using NaCl counter ions. The complex underwent an initial 1000-step energy minimization, followed by 100 ps of NVT and NPT equilibration. Subsequently, MD simulations were conducted for 100 ns for each system, utilizing periodic boundary conditions and maintaining them at 310 K and 1.0 bar. The minimum distance of 1.0 nm was established between the simulation box and the protein along the XYZ axes. A 2 fs time step was applied, and energy minimization was performed using the steepest descent algorithm, with a 1.4 nm cutoff utilized for both Coulomb and van der Waals interactions.

## 5. Conclusions

This study utilized UHPLC-Q-TOF-MS/MS for chemical characterization, followed by network pharmacology leveraging anti-allergic drug target information, molecular docking, and MD simulation, to systematically explore the potentially effective compounds and mechanisms of ACP. Our findings reveal a dual mechanistic profile: it is hypothesized that key compounds such as M12 may act on core targets such as MAPK1 to activate pathways like the PI3K-Akt signaling pathway, thereby exerting distinctive effects that differ from the mechanisms of existing anti-allergic drugs. Concurrently, other key compounds such as M90 may act on core targets like ADRA2A to activate pathways such as the cGMP-PKG signaling pathway, thereby exerting an effect similar to certain existing anti-allergic drugs. This comprehensive analysis fully reflects the multi-compound, multi-target, and multi-pathway regulatory impact characteristic of TCM, laying a robust computational foundation for further in-depth experimental research. It should be noted that the therapeutic mechanism of ACP for allergic diseases was currently predicted via network pharmacology, but lack of experimental verification is the limitation. Therefore, comprehensive experimental validation, including in vitro and in vivo studies, is indispensable to confirm the proposed mechanisms and the anti-allergic effects of ACP in future investigations.

## Figures and Tables

**Table 1:** The core targets obtained from the key pathway-target network.

No.-Unique	Gene Nane	Degree	No.-Common	Gene Nane	Degree
1	Mitogen-activated protein kinase 1 (MAPK1)	9	1	Alpha-1D adrenergic receptor (ADRA1D)	4
2	Mitogen-activated protein kinase 3 (MAPK3)	9	2	Beta-1 adrenergic receptor (ADRB1)	4
3	Phosphatidylinositol 4,5-bisphosphate 3-kinase catalytic subunit delta isoform (PIK3CD)	9	3	Alpha-1A adrenergic receptor (ADRA1A)	4
4	Phosphatidylinositol 4,5-bisphosphate 3-kinase catalytic subunit beta isoform (PIK3CB)	9	4	Alpha-2B adrenergic receptor (ADRA2B)	1
5	RAC-alpha serine/threonine-protein kinase (AKT1)	8	5	Alpha-2A adrenergic receptor (ADRA2A)	1
6	Phosphatidylinositol 4,5-bisphosphate 3-kinase catalytic subunit alpha isoform (PIK3CA)	8	6	Hematopoietic prostaglandin D synthase (HPGDS)	1
7	Phosphatidylinositol 3-kinase regulatory subunit alpha (PIK3R1)	8	7	Polyunsaturated fatty acid 5-lipoxygenase (ALOX5)	1
8	Transcription factor p65 (RELA)	8	8	Thromboxane-A synthase (TBXAS1)	1
9	Nuclear factor NF-kappa-B p105 subunit (NFKB1)	8	9	Thromboxane A2 receptor (TBXA2R)	1
10	Mitogen-activated protein kinase 8 (MAPK8)	8			

**Table 2:** The key compounds obtained from the core target-compound network.

No.-Unique	Mol ID	Degree	Compounds	No.-Common	Mol ID	Degree	Compounds
1	M12	7	Lyoniresinol	1	M90	5	Naringin-3-O-(3-hydroxy-3-methylglutarate)-glucoside
2	M54	5	Limonin	2	M93	4	Glycyrrhizic acid
3	M26	5	7-Acetoxy-2-methylisoflavone	3	M20	4	Heptopyranosides
4	M92	4	Apiole	4	M89	4	Licorice-saponin G2
5	M88	4	Phellopterin	5	M94	3	Uralsaponin A
6	M87	4	Byakangelicol	6	M11	3	Phenylalanine
7	M30	3	Caffeic acid	7	M17	2	Tryptophan
8	M64	2	Nomilin	8	M73	2	Diosmin
9	M71	2	Naringenin	9	M50	2	Luteolin-7-O-β-D-glucoside
10	M45	2	Dillapiol	10	M59	2	Hyperoside
11	M86	2	Nortangeretin	11	M55	2	Leucoside
12	M80	2	Luteolin	12	M65	2	Kaempferol 3-O-robinobioside
13	M81	2	Kaempferol	13	M57	2	Rutin
14	M85	2	Quercetin	14	M72	2	Genistein
				15	M82	2	Daidzein

## Data Availability

The original contributions presented in this study are included in the article/Supplementary Material. Further inquiries can be directed to the corresponding authors.

## Supplementary Materials

The following supporting information can be downloaded at: https://www.mdpi.com/article/10.3390/ph18101444/s1, Figure S1: Base peak chromatograms (BPC) of ACP in positive ion mode; Figure S2: The fragmentation pathway of flavonoids (A-Daidzin (No. **62**), B-Puerarin (No. **29**)); Figure S3: The fragmentation pathway of tyrosine (No. **10**).; Figure S4: The fragmentation pathway of regaloside A (No. **48**); Figure S5: The fragmentation pathway of glycyrrhizic acid (No. **123**); Figure S6: The fragmentation pathway of nucleosides (A-Adenosine (No. **11**), B-Guanosine (No. **17**), C-Uridine (No. **14**)); Figure S7: The fragmentation pathway of sweroside (No. **47**); Table S1: Identification of chemical constituents from ACP by UPLC-Q-TOF-MS; Table S2: The key unique pathways in ACP for the treatment of allergic diseases; Table S3: The key common pathways in ACP for the treatment of allergic diseases; Table S4: Molecular docking results regarding the unique part (kcal/mol); Table S5: Molecular docking results regarding the common parts (kcal/mol).

## Abbreviations

The following abbreviations are used in this manuscript:

TCM	Traditional Chinese Medicine
ACP	Allergic constitution prescription
UHPLC-Q-TOF-MS/MS	Ultra-high performance liquid chromatography quadrupole time-of-flight tandem mass spectrometry
KEGG	Kyoto Encyclopedia of Genes and Genomes
WAO	World Allergy Organization
WHO	World Health Organization
MD	Molecular dynamics
RMSD	Root mean squared deviation
Rg	Radius of gyration
RMSF	Root mean squared fluctuations
SASA	Solvent accessible surface area
QAMS	Quantitative analysis of multi-components by single-marker
DRS	Digital reference standard
TRSDMC	Two reference substances for determination of multiple components
QASRE	Quantitative analysis by standardized reference extract
