# Supplementary material for: Characterization and Anti-Allergic Mechanisms of Bioactive Compounds in a Traditional Chinese Medicine Prescription Using UHPLC-Q-TOF-MS/MS, Network Pharmacology and Computational Simulations
Source: Pharmaceuticals (Basel). 2025 Sep 26;18(10):1444. doi: 10.3390/ph18101444 (PMC12566646; doi:10.3390/ph18101444)
Supplement: Supplementary file 1 [file pharmaceuticals-18-01444-s001.zip › pharmaceuticals-3859274-supplementary.pdf]

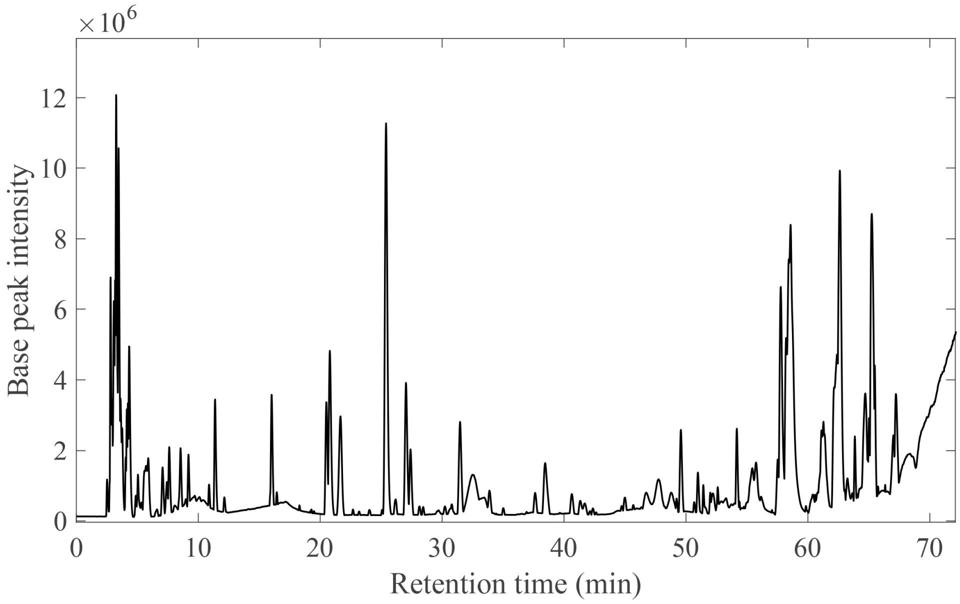

Figure S1. Base peak chromatograms (BPC) of ACP in positive ion mode.

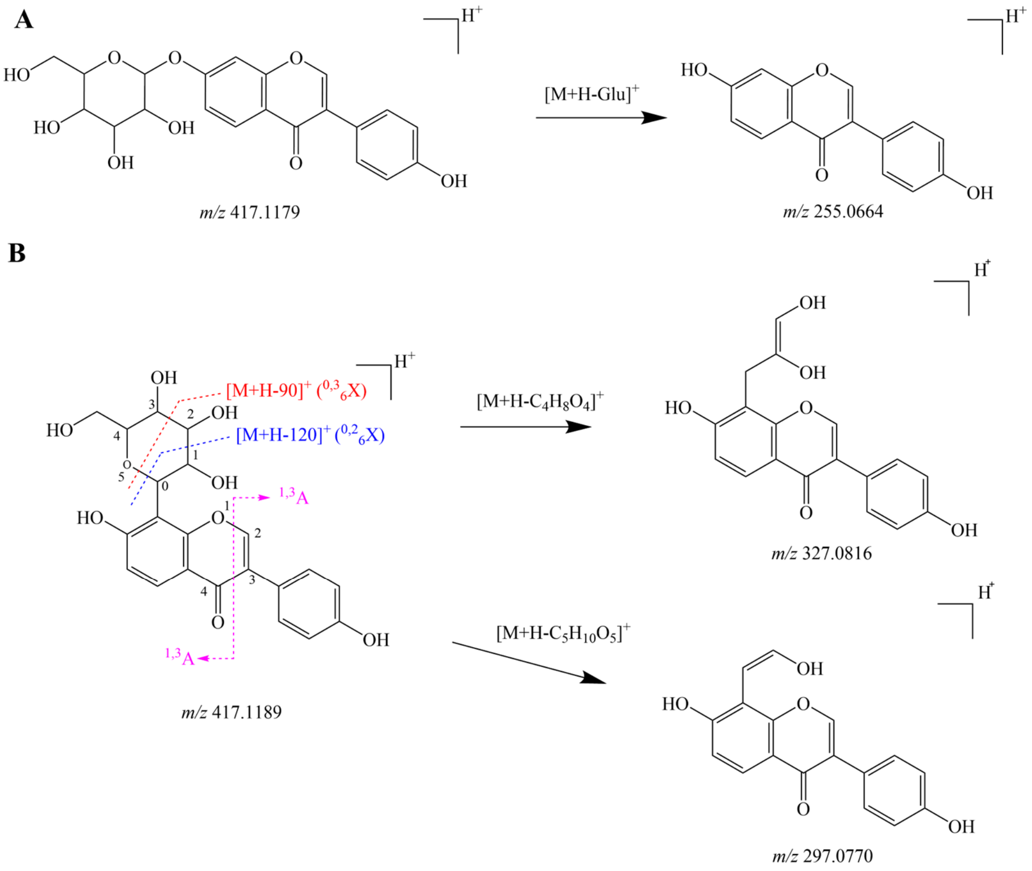

Figure S2. The fragmentation pathway of flavonoids (A-Daidzin (No. **62**), B-Puerarin (No. **29**)).

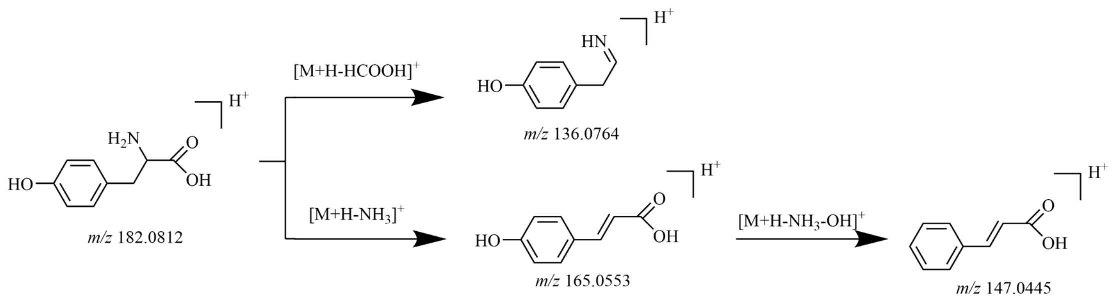

Figure S3. The fragmentation pathway of tyrosine (No. **10**).

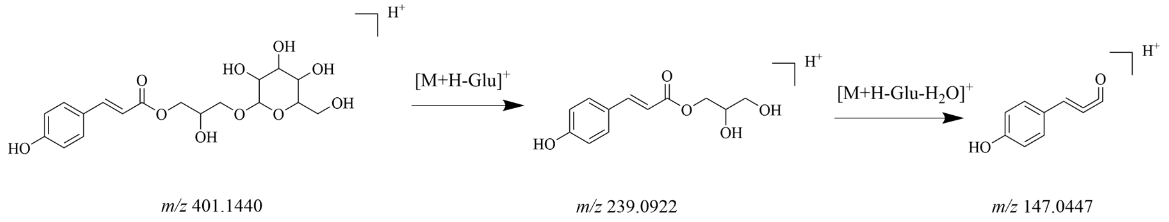

Figure S4. The fragmentation pathway of regaloside A (No. **48**).

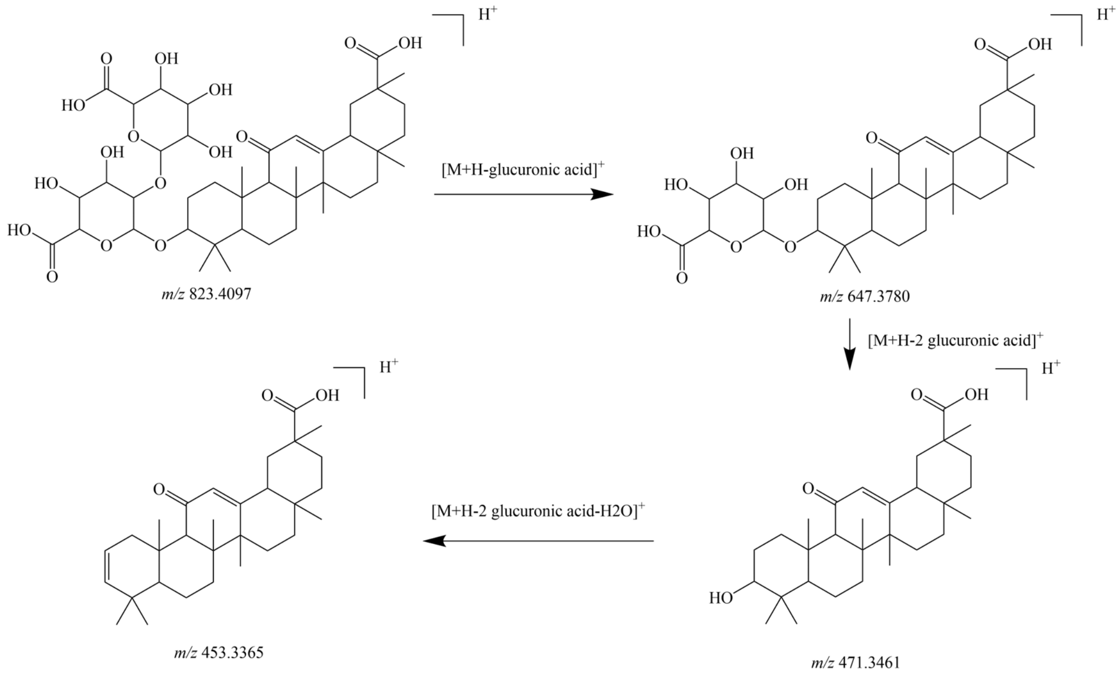

Figure S5. The fragmentation pathway of glycyrrhizic acid (No. **123**).

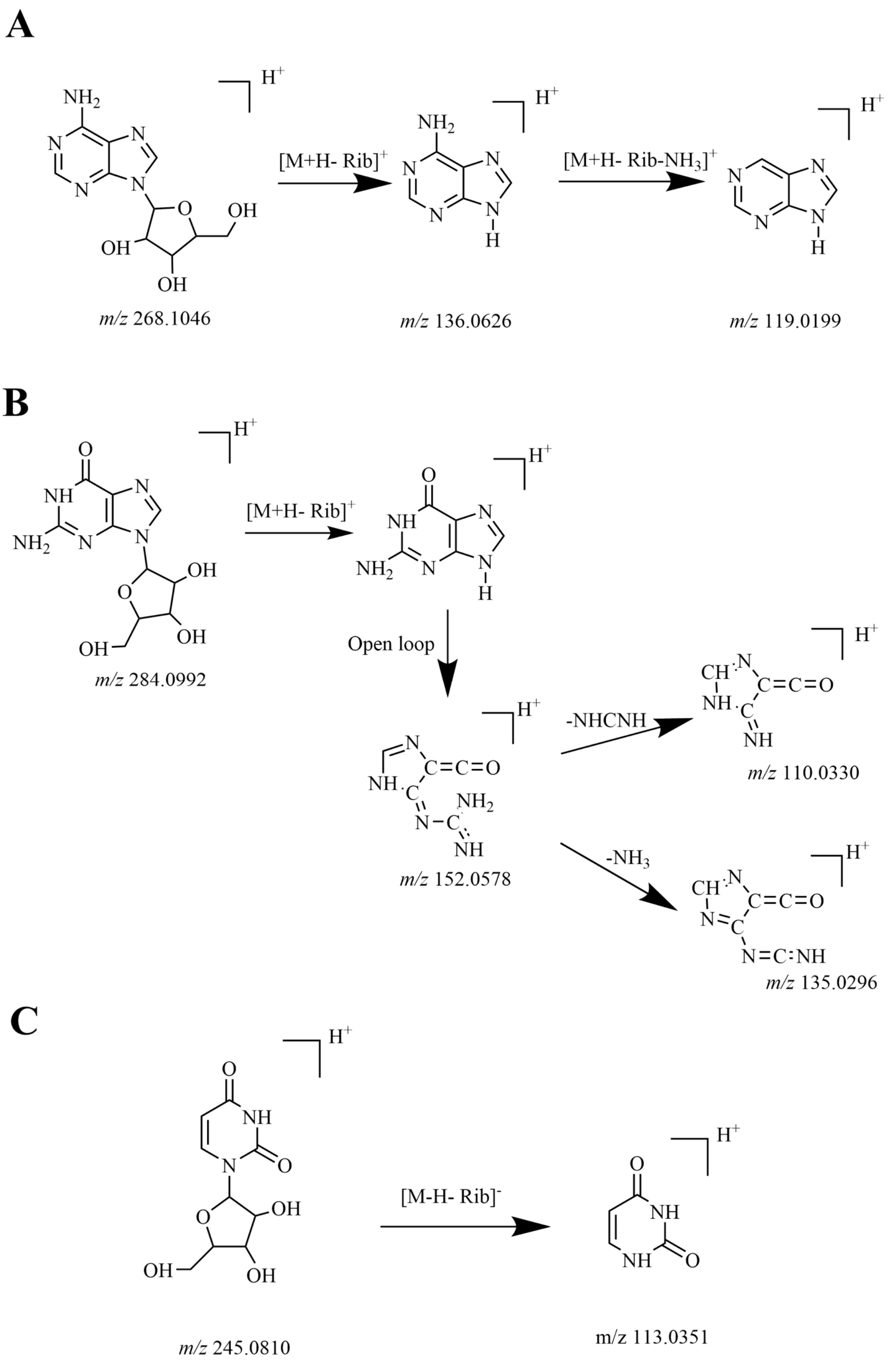

Figure S6. The fragmentation pathway of nucleosides (A-Adenosine (No. **11**), B-Guanosine (No. **17**), C-Uridine (No. **14**)).

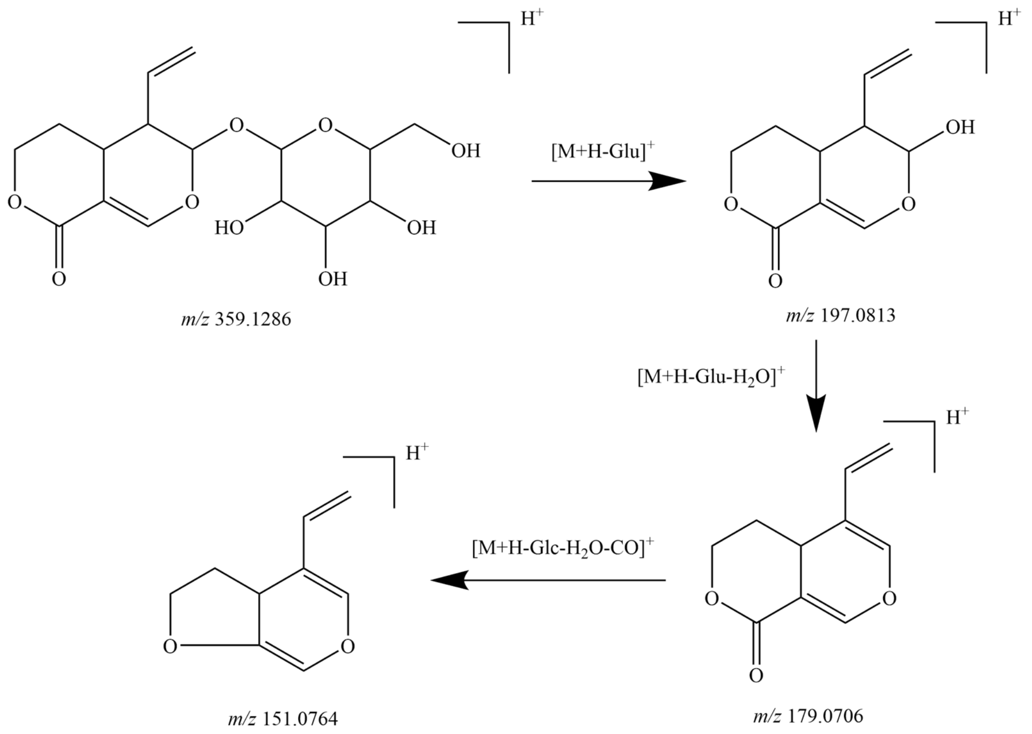

Figure S7. The fragmentation pathway of sweroside (No. **47**).

Table S1. Identification of chemical constituents from ACP by UPLC-Q-TOF-MS.

No.-peak	No.-network	Rt (min)	Formula	Adducts	Cal (*m/z*)	Observed (*m/z*)	Mass error (ppm)	Collision energy (eV)	MS^2 fragment ions (*m/z*)	Identification	Type
1	M1	3.1	$C_7H_{13}NO_3$	$[M+H]^+$	160.0968	160.0978	-1.0	20	114.0921, 88.0761	N-Isovaleroylglycine or isomers	A [a]
2	M2	3.4	$C_6H_9NO_3$	$[M+H]^+$	144.0655	144.0657	-0.1	30	102.0559, 84.0816	6-Oxopiperidine-2-carboxylic acid	A
3	M3	3.5	$C_{11}H_{19}NO_7$	$[M+H]^+$	278.1324	278.1241	-0.7	20	260.1142, 242.1034, 232.1187, 214.1081, 128.0714	N-(1-Deoxy-D-fructose-1-yl)-L-proline	A
4	M1	3.7	$C_7H_{13}NO_3$	$[M+H]^+$	160.0968	160.0671	-0.3	15	142.0508, 114.0559, 96.0452	N-Isovaleroylglycine or isomers	A
5	M4	4.5	$C_5H_7NO_3$	$[M+H]^+$	130.0499	130.0506	-0.5	20	102.0561, 84.0448	Pyroglutamic acid or isomers	A
6	M5	4.7	$C_9H_{11}NO$	$[M+H]^+$	150.0913	150.0919	-0.6	20	135.0684, 119.0497, 107.0499, 91.0547	Cathinone or isomers	O [o]
7	M4	4.9	$C_5H_7NO_3$	$[M+H]^+$	130.0499	130.0509	-0.7	20	110.0332, 84.0450	Pyroglutamic acid or isomers	A
8	M6	5.7	$C_9H_{11}NO_3$	$[M+H]^+$	182.0812	182.0818	-0.2	15	165.0554, 147.0449, 136.0764, 123.0448, 121.0654, 119.0498	Tyrosine or isomers	A
9	M4	5.8	$C_5H_7NO_3$	$[M+H]^+$	130.0499	130.0511	-1.3	20	113.9645, 102.0540, 84.0450	Pyroglutamic acid or isomers	A
10	M6	5.9	$C_9H_{11}NO_3$	$[M+H]^+$	182.0812	182.0831	-2.0	20	165.0553, 147.0445, 136.0764, 123.0446, 91.0547	Tyrosine or isomers	A

11	M7	6.5	$C_{10}H_{13}N_5O_4$	$[M+H]^+$	268.1040	268.1046	-0.6	15	136.0626, 119.0199	Adenosine or isomers	N [n]
12	M8	7.0	$C_6H_{13}NO_2$	$[M+H]^+$	132.1019	132.1031	-1.2	20	86.0969	Leucine or isomers	A
13	M7	7.1	$C_{10}H_{13}N_5O_4$	$[M+H]^+$	268.1040	268.1047	-0.7	15	136.0626	Adenosine or isomers	N
14	M9	7.2	$C_9H_{12}N_2O_6$	$[M+H]^+$	245.0768	245.0810	-1.7	15	113.0351	Uridine or isomers	N
15	M7	7.4	$C_{10}H_{13}N_5O_4$	$[M+H]^+$	268.1040	268.1048	-0.8	15	136.0626	Adenosine or isomers	N
16	M8	7.5	$C_6H_{13}NO_2$	$[M+H]^+$	132.1019	132.1017	0.2	20	113.9665, 98.5127, 86.0973	Leucine or isomers	A
17	M10	7.8	$C_{10}H_{13}N_5O_5$	$[M+H]^+$	284.0989	284.0992	-0.2	10	152.0575	Guanosine or isomers	N
18	M10	8.4	$C_{10}H_{13}N_5O_5$	$[M+H]^+$	284.0989	284.0990	0.0	10	152.0578	Guanosine or isomers	N
19	M7	8.6	$C_{10}H_{13}N_5O_4$	$[M+H]^+$	268.1040	268.1048	-0.8	15	136.0627	Adenosine or isomers	N
20	M11	11.3	$C_9H_{11}NO_2$	$[M+H]^+$	166.0863	166.0868	-0.6	15	120.0817, 103.0547	Phenylalanine	A
21	M12	12.4	$C_{22}H_{28}O_8$	$[M+H]^+$	421.1857	421.1690	3.9	25	289.0913, 199.0719, 127.0393	Lyoniresinol	O
22	M4	13.7	$C_5H_7NO_3$	$[M+H]^+$	130.0863	130.0875	-1.5	20	98.5120, 85.0288	Pyroglutamic acid or isomers	A
23	M13	14.6	$C_{22}H_{28}O_{14}$	$[M+H]^+$	517.1552	517.1547	0.5	15	325.0913, 163.0393	4-*O*-(3'-*O*-α-D-Glucopyranosyl)-caffeoyl quinic acid or isomers	PA [pa]
24	M13	14.9	$C_{22}H_{28}O_{14}$	$[M+H]^+$	517.1552	517.1538	1.4	15	325.0920, 163.0395	4-*O*-(3'-*O*-α-D-Glucopyranosyl)-caffeoyl quinic acid or isomers	PA
25	M14	15.5	$C_{21}H_{18}O_6$	$[M+H]^+$	367.1176	367.1191	-4.1	20	349.1390, 303.1339, 229.0973	Glycyrol	O
26	M15	15.9	$C_{16}H_{18}O_9$	$[M+H]^+$	355.1024	355.1027	-0.3	10	163.0399	Chlorogenic acid	PA

27	M16	16.0	$C_{30}H_{48}O_5$	$[M+H]^+$	489.2424	489.2410	1.5	15	256.1162	Tormentic acid	O
28	M17	16.4	$C_{11}H_{12}N_2O_2$	$[M+H]^+$	205.0972	205.0981	-0.7	10	188.0717, 161.0817	Tryptophan	A
29	M18	16.9	$C_{21}H_{20}O_9$	$[M+H]^+$	417.1180	417.1189	-0.2	30	327.0861, 297.0770, 253.1546	Puerarin	F [f]
30	M19	18.1	$C_{17}H_{24}O_{11}$	$[M+H]^+$	405.1391	405.1397	-0.6	10	243.0875, 211.0612	Secoxyloganin or isomers	I [I]
31	M20	18.2	$C_{13}H_{24}O_7$	$[M+H]^+$	293.1595	293.1612	-1.6	25	234.1127, 163.0396, 114.1031	Heptopyranosides	O
32	M21	18.7	$C_7H_6O_3$	$[M+H]^+$	139.0390	139.0395	-0.6	20	120.0459, 111.0448, 93.0342	4-Hydroxybenzoic acid	PA
33	M22	19.6	$C_{27}H_{30}O_1$	$[M+H]^+$	579.1708	579.1695	1.3	25	417.1184, 255.0661	Daidzein-4`,7-*O*-glucoside	F
34	M23	20.8	$C_{16}H_{18}O_9$	$[M+H]^+$	355.1024	355.1030	-0.6	10	163.0400	Isochlorogenic acid	PA
35	M24	21.1	$C_{28}H_{32}O_{15}$	$[M+H]^+$	609.1814	609.1825	-1.0	30	447.1282, 285.0762	3`-Methyoxy-4`-*O*-glucosyl-daidzin	F
36	M25	21.5	$C_{16}H_{22}O_{10}$	$[M+H]^+$	375.1386	375.1255	2.9	20	213.0762, 195.0656, 177.0551, 151.0395, 125.0239, 107.0496	Secologanic acid or isomer	I
37	M25	21.7	$C_{16}H_{22}O_{10}$	$[M+H]^+$	375.1386	375.1260	2.6	15	213.0767, 195.0660, 177.0551, 151.0396, 125.0240, 107.0498	Secologanic acid or isomer	I
38	M19	22.0	$C_{17}H_{24}O_{11}$	$[M+H]^+$	405.1391	405.1393	-0.2	10	243.0871, 211.0608, 167.0344	Secoxyloganin or isomers	I
39	M26	22.5	$C_{18}H_{14}O_4$	$[M+H]^+$	295.0965	295.0891	2.5	20	261.0987, 215.0919, 157.0503, 145.0498, 133.0505, 115.0396, 97.0290, 85.0300	7-Acetoxy-2-methylisoflavone	F
40	M27	22.7	$C_{26}H_{28}O_{14}$	$[M+H]^+$	565.1552	565.1540	1.1	33	433.1129, 415.1020, 397.0914,	3`-Hydroxypuerarin xyloside	F

									367.0810, 337.0702, 313.0708		
41	M28	23.4	$C_{27}H_{30}O_{14}$	$[M+H]^+$	579.1708	579.1690	1.8	35	417.1182, 399.1s071, 381.0975, 363.0868, 351.0866, 321.0759, 297.0760, 279.0645, 267.0646, 257.0810, 239.0699, 214.0845	Puerarin-4`-*O*-*β*-D-glucoside or isomers	F
42	M29	23.5	$C_{10}H_8O_3$	$[M+H]^+$	177.0546	177.0552	-0.6	20	160.0762, 145.0288, 125.0036, 117.0337, 106.9929, 89.0389	2-Formylcinnamic acid	O
43	M30	24.1	$C_9H_8O_4$	$[M+H]^+$	181.0495	181.0497	-0.1	15	163.0396, 145.0289, 135.0444, 117.0343, 107.0495, 89.0391	Caffeic acid	PA
44	M31	24.3	$C_{26}H_{28}O_{13}$	$[M+H]^+$	549.1603	549.1594	0.8	33	417.1174, 399.1069, 381.0963, 363.0866, 351.0860, 297.0754	6``-*O*-Xylosylpuerarin	F
45	M28	24.7	$C_{27}H_{30}O_{14}$	$[M+H]^+$	579.1708	579.1696	1.2	35	417.1177, 399.1069, 381.0965, 363.0862, 351.0864, 327.0866, 321.0755, 297.0759	Puerarin-4`-*O*-*β*-D-glucoside or isomers	F
46	M32	25.2	$C_{21}H_{20}O_{10}$	$[M+H]^+$	433.1129	433.1120	1.6	15	271.0606	Genistin or isomers	F
47	M33	26.7	$C_{16}H_{22}O_9$	$[M+H]^+$	359.1337	359.1286	1.4	10	197.0813, 179.0706, 151.0764, 127.0392	Sweroside	I
48	M34	28.1	$C_{18}H_{24}O_{10}$	$[M+H]^+$	401.1442	401.1440	0.2	10	309.0974, 239.0920, 147.0447	Regaloside A or isomers	PA
49	M35	28.3	$C_{18}H_{24}O_{10}$	$[M+H]^+$	401.1442	401.1436	0.5	10	239.0922, 147.0447	Regaloside D or isomers	PA

50	M36	29.3	$C_{28}H_{32}O_{16}$	$[M+H]^+$	625.1763	625.1767	-0.4	30	607.1642, 589.1533, 571.1443, 559.1446, 553.1319, 541.1321, 535.1231, 529.1341, 511.1239, 505.1327, 487.1231, 469.1117, 457.1128, 451.1016, 439.1043, 421.0911, 409.0916, 385.0924, 379.0814, 367.0828, 355.0819, 337.0691, 319.0809	6,8-Di-*C*-glucosyldiosmetin	F
51	M37	29.4	$C_{27}H_{32}O_{14}$	$[M+H]^+$	581.1865	581.1863	0.0	20	273.0761, 129.0554	Naringin	F
52	M38	29.4	$C_{27}H_{30}O_{14}$	$[M+H]^+$	579.1708	579.1742	-0.6	20	435.1287, 419.1328, 401.1233, 383.1123, 365.1029, 315.0875, 273.0767, 147.0663, 129.0555, 85.0295	Rhoifolin	F
53	M39	29.4	$C_{27}H_{26}O_{18}$	$[M+H]^+$	639.1192	639.1173	0.3	25	463.0856, 287.0552	Luteolin 7-*O*-diglucuronide or isomers	F
54	M40	29.4	$C_{33}H_{42}O_{19}$	$[M+H]^+$	743.2393	743.2409	-0.2	20	621.1804, 597.1780, 581.1857, 563.1751, 545.1665, 527.1491, 501.1393, 477.1411, 459.1288, 435.1290, 419.1332, 401.1240, 383.1104, 339.0872, 315.0858, 273.0761, 231.0843, 217.0701, 199.0633, 163.0618, 147.0661	Narirutin 4'-glucoside	F
55	M39	29.5	$C_{27}H_{26}O_{18}$	$[M+H]^+$	639.1192	639.1183	0.2	20	463.0864, 287.0556	Luteolin 7-O-diglucuronide or	F

										isomers	
56	M41	29.6	$C_{27}H_{32}O_{14}$	$[M+H]^+$	581.1865	581.1873		20	435.1277, 273.0762, 129.0554	Narirutin	F
57	M42	29.8	$C_{17}H_{26}O_{10}$	$[M+H]^+$	391.1599	391.1598	0.0	10	229.1079, 211.0972, 197.0814, 179.0709	Loganin	O
58	M43	30.0	$C_{26}H_{28}O_{13}$	$[M+H]^+$	549.1603	549.1582	0.4	20	417.1180, 255.0653	Ambonin	F
59	M22	30.2	$C_{27}H_{30}O_{14}$	$[M+H]^+$	579.1708	579.1692	0.3	20	255.0658	Daidzein-4`,7-*O*-glucoside	F
60	M44	30.4	$C_{22}H_{22}O_{10}$	$[M+H]^+$	447.1286	447.1279	0.2	25	411.1065, 381.0963, 359.1313, 327.0867, 297.0755, 215.0926, 103.0394	3`-Methoxypuerarin	F
61	M45	30.7	$C_{12}H_{14}O_4$	$[M+H]^+$	223.0965	223.0957	-0.4	15	193.0500, 165.0551, 151.0397	Dillapiol or isomers	O
62	M46	31.6	$C_{21}H_{20}O_9$	$[M+H]^+$	417.1180	417.1179	0.1	20	255.0664	Daidzin	F
63	M47	32.6	$C_{17}H_{24}O_{10}$	$[M+H]^+$	389.1442	389.1429	1.3	10	371.1331, 357.1181, 343.1148, 325.1045, 315.1199, 269.0796, 251.0693, 227.0923, 209.0816, 195.0657, 177.0550, 165.0553, 151.0395, 107.0497	Epi-vogeloside	O
64	M48	33.9	$C_{22}H_{22}O_{10}$	$[M+H]^+$	447.1286	447.1279	0.2	15	285.0764	3`-Methoxydaidzin or isomer	F
65	M49	35.9	$C_{27}H_{32}O_{15}$	$[M+H]^+$	597.1814	597.1463	5.8	15	465.1009, 435.0936, 303.0494, 133.0499	Viscumneoside III	F
66	M50	36.2	$C_{21}H_{20}O_{11}$	$[M+H]^+$	449.1079	449.1251	3.8	25	287.0552	Luteolin-7-*O*-*β*-D-glucoside	F

67	M51	36.5	$C_{29}H_{34}O_{14}$	$[M+H]^+$	607.2021	607.2015	0.1	10	461.1446, 299.0926	Pueroside A	F
68	M52	36.8	$C_{21}H_{22}O_9$	$[M+H]^+$	419.1337	419.1332	0.1	10	257.0818	Liquiritin or isomers	F
69	M53	37.7	$C_{18}H_{26}O_{10}$	$[M+H]^+$	403.1599	403.1608	-0.2	10	241.1086, 223.0978, 195.0666, 177.0557, 151.0400, 107.0500	7-*O*-ethyl sweroside or isomer	F
70	M54	38.1	$C_{26}H_{30}O_8$	$[M+H]^+$	471.2013	471.2083	-6.9	10	229.1127	Limonin	O
71	M55	38.2	$C_{26}H_{28}O_{15}$	$[M+H]^+$	581.1501	581.1474	0.5	25	449.1066, 287.0550	Leucoside	F
72	M52	38.6	$C_{21}H_{22}O_9$	$[M+H]^+$	419.1337	419.1352	-0.4	10	257.0822	Liquiritin or isomers	F
73	M56	38.7	$C_{26}H_{30}O_{13}$	$[M+H]^+$	551.1759	551.1744	0.3	10	419.1345, 257.0819	Liquiritin-apioside or isomers	F
74	M57	39.4	$C_{27}H_{30}O_{16}$	$[M+H]^+$	611.1607	611.1597	0.2	15	465.1032, 449.1085, 303.0509, 147.0662, 129.0557, 85.0283	Rutin	F
75	M58	39.8	$C_{27}H_{30}O_{15}$	$[M+H]^+$	595.1657	595.1593	1.1	25	463.1234, 449.1074, 287.0560, 129.0536	3`-Hydroxy-4`-*O*-*β*-D-glucosyl-Puerarin	F
76	M59	40.4	$C_{21}H_{20}O_{12}$	$[M+H]^+$	465.1028	465.1012	0.3	10	303.0505	Hyperoside	F
77	M53	40.7	$C_{18}H_{26}O_{10}$	$[M+H]^+$	403.1599	403.1603	-0.1	10	241.1083, 223.0977, 195.0664, 177.0554, 151.0397, 107.0497	7-*O*-ethyl sweroside or isomer	O
78	M60	41.0	$C_{26}H_{28}O_{14}$	$[M+H]^+$	565.1552	565.1552	0.0	20	433.1125, 271.0605, 133.0499	Genistein-8-*C*-apiosyl(1→6)-glucoside	F
79	M61	42.1	$C_{21}H_{18}O_{12}$	$[M+H]^+$	463.0871	463.0870	0.1	20	287.0559	Luteolin 7-glucuronide	F
80	M32	42.5	$C_{21}H_{20}O_{10}$	$[M+H]^+$	433.1129	433.1126	0.3	20	399.1060, 271.0610	Genistin or isomers	F

81	M62	42.5	$C_{30}H_{36}O_{15}$	$[M+H]^+$	637.2127	637.2120	0.7	15	475.1598, 313.1077	Pueroside B	O
82	M63	42.8	$C_{21}H_{18}O_{12}$	$[M+H]^+$	463.0871	463.0875	0.0	20	445.1479, 427.1955, 409.1846, 331.1758, 301.1652, 283.1545, 265.1438, 237.1496, 219.1394, 187.1337, 169.1231, 151.1125, 123.1176, 97.0291	Tectoridin	F
83	M41	45.2	$C_{27}H_{32}O_{14}$	$[M+H]^+$	581.1865	581.1856	0.9	15	435.1290, 419.1340, 401.1233, 383.1125, 339.0868, 315.0866, 273.0768, 263.0557, 255.0878, 237.0755, 195.0301, 163.0607, 147.0661, 129.0554, 85.0291	Narirutin or isomers	F
84	M64	45.6	$C_{29}H_{22}O_9$	$[M+H]^+$	515.1337	515.1321	1.6	20	499.1237, 425.1378, 355.1046, 337.0926, 319.0827, 163.0401	Nomilin	O
85	M65	45.8	$C_{27}H_{30}O_{15}$	$[M+H]^+$	595.1657	595.1676	-0.3	20	449.1088, 287.0557, 147.0658	Kaempferol 3-*O*-robinobioside	F
86	M66	46.8	$C_{27}H_{30}O_{14}$	$[M+H]^+$	579.1708	579.1706	0.2	20	433.1130, 271.0610	6-Methoxy-7-xylosyl-genistin	F
87	M36	47.5	$C_{28}H_{32}O_{16}$	$[M+H]^+$	625.1763	625.1755	0.8	15	479.1178, 317.0661, 147.0659, 129.0551	6,8-Di-*C*-glucosyldiosmetin isomers	F
88	M67	47.7	$C_{25}H_{24}O_{12}$	$[M+H]^+$	517.1341	517.1337	0.3	10	499.1240, 337.0924, 163.0404	Dicaffeoylquinic acid or isomer (1,3-Dicaffeoylquinic acid; 3,4-Dicaffeoylquinic acid; 3,5-O-dicaffeoylquinic acid; 4,5-	PA

										Dicaffeoylquinic acid)	
89	M45	48.8	$C_{12}H_{14}O_4$	$[M+H]^+$	223.0965	223.0978	-1.0	20	205.0864, 181.0864, 171.0298, 153.0192, 121.0663	Dillapiol	O
90	M71	49.1	$C_{15}H_{12}O_5$	$[M+H]^+$	273.0757	273.0754	0.4	25	153.0186, 147.0443, 119.0496	Naringenin	F
91	M72	49.2	$C_{15}H_{10}O_5$	$[M+H]^+$	271.0601	271.0601	0.0	30	253.0497, 243.0644, 225.0545, 215.0705, 197.0604, 187.0750, 169.0655, 161.0228, 153.0185, 147.0450, 137.0244, 119.0496, 91.0550	Genistein or isomer	F
92	M73	49.7	$C_{28}H_{32}O_{15}$	$[M+H]^+$	609.1814	609.1781	1.0	25	465.1384, 449.1435, 431.1330, 413.1226, 395.1114, 369.0965, 345.0968, 303.0870, 129.0552	Diosmin	F
93	M68	50.7	$C_{25}H_{24}O_{12}$	$[M+H]^+$	517.1341	517.1338	0.3	10	499.1228, 417.1069, 355.1022, 337.0916, 309.0967, 251.0679, 163.0397	Dicaffeoylquinic acid or isomer (1,3-Dicaffeoylquinic acid; 3,4-Dicaffeoylquinic acid; 3,5-O-dicaffeoylquinic acid; 4,5-Dicaffeoylquinic acid)	PA
94	M69	50.8	$C_{25}H_{24}O_{12}$	$[M+H]^+$	517.1341	517.1334	0.6	10	499.1228, 417.1059, 355.1018, 337.0917, 309.0962, 279.0996, 251.0682, 163.0397	Dicaffeoylquinic acid or isomer (1,3-Dicaffeoylquinic acid; 3,4-Dicaffeoylquinic acid; 3,5-O-Dicaffeoylquinic acid; 4,5-Dicaffeoylquinic acid)	PA

95	M74	51.1	$C_{23}H_{22}O_{10}$	$[M+H]^+$	459.1286	459.1287	-0.1	20	255.0662	6``-O-Acetyl daidzin	F
96	M75	51.3	$C_{27}H_{30}O_{13}$	$[M+H]^+$	563.1759	563.1751	0.8	20	431.1338, 269.0814	Glycyroside	F
97	M76	52.1	$C_{24}H_{26}O_{10}$	$[M+H]^+$	475.1599	475.1601	-0.2	20	313.1084, 219.0662, 107.0500	Sophoraside A	O
98	M77	52.4	$C_{10}H_{12}O_4$	$[M+H]^+$	197.0808	197.0817	-0.9	20	182.0575, 169.0865, 154.0633, 138.0682, 123.0815, 106.9926	Sweroside aglycone	O
99	M78	52.6	$C_{26}H_{30}O_{13}$	$[M+H]^+$	551.1759	551.1740	1.9	15	419.1333, 257.0812, 133.0503	Isoliquiritin apioside	F
100	M79	52.7	$C_{22}H_{22}O_9$	$[M+H]^+$	431.1337	431.1344	-0.7	15	269.0823	Ononin	F
101	M52	52.9	$C_{21}H_{22}O_9$	$[M+H]^+$	419.1337	419.1333	0.4	15	257.0817	Liquiritin or isomer	F
102	M80	53.3	$C_{15}H_{10}O_6$	$[M+H]^+$	287.0550	287.0565	-1.4	50	269.0450, 223.0355, 203.0342, 173.0239, 147.0442, 129.0340, 89.0386	Luteolin or Kaempferol	F
103	M52	53.4	$C_{21}H_{22}O_9$	$[M+H]^+$	419.1337	419.1339	-0.2	15	257.0816	Liquiritin or isomer	F
104	M82	54.2	$C_{15}H_{10}O_4$	$[M+H]^+$	255.0652	255.0657	-0.5	40	237.0553, 227.0710, 199.0763, 181.0657, 171.0811, 153.0703, 137.0241, 91.0548	Daidzein	F
105	M83	54.8	$C_{12}H_8O_5$	$[M+H]^+$	233.0444	233.0446	-0.2	30	218.0213, 190.0266, 162.0318, 134.0366	8-Hydroxybergapten	O
106	M84	54.9	$C_{27}H_{30}O_{14}$	$[M+H]^+$	579.1708	579.1683	2.5	20	447.1281, 285.0759, 133.0501	Isorhoifolin	F
107	M85	55.3	$C_{15}H_{10}O_7$	$[M+H]^+$	303.0499	303.0511		20	203.0344, 159.0445, 147.0445, 131.0496, 91.0546	Quercetin	F

108	M70	55.4	$C_{25}H_{24}O_{12}$	$[M+H]^+$	517.1341	517.1330	1.1	25	269.0814, 131.0344	Dicaffeoylquinic acid or isomer (1,3-Dicaffeoylquinic acid; 3,4-Dicaffeoylquinic acid; 3,5-O-dicaffeoylquinic acid; 4,5-Dicaffeoylquinic acid)	PA
109	M81	55.7	$C_{15}H_{10}O_6$	$[M+H]^+$	287.0550	287.0553	-0.3	50	269.0456, 253.0514, 241.0507, 225.0554, 213.0555, 197.0614, 185.0606, 161.0247, 153.0191, 135.0449, 117.0341, 105.0344, 89.0394	Luteolin or Kaempferol	F
110	M86	55.9	$C_{15}H_{10}O_7$	$[M+H]^+$	303.0499	303.0500	-0.6	30	229.0502, 153.0186	Nortangeretin	F
111	M87	56.0	$C_{17}H_{16}O_6$	$[M+H]^+$	317.1020	317.1025	-0.6	20	273.0766, 231.0294	Byakangelicol	O
112	M48	56.5	$C_{22}H_{22}O_{10}$	$[M+H]^+$	447.1286	447.1284	0.1	15	285.0763, 131.0349	3`-Methoxydaidzin or isomer	F
113	M88	56.6	$C_{17}H_{16}O_5$	$[M+H]^+$	301.1071	301.1078	-0.8	20	197.0456, 131.0498	Phellopterin	~~O~~
114	M89	57.0	$C_{42}H_{62}O_{17}$	$[M+H]^+$	839.4060	839.4061	-0.2	15	663.3738, 469.3312	Licorice-saponin G2 or isomers	S
115	M90	57.1	$C_{33}H_{40}O_{18}$	$[M+H]^+$	725.2287	725.2274	1.3	25	419.1342	Naringin-3-*O*-(3-hydroxy-3-methylglutarate)-glucoside	F
116	M91	57.2	$C_{44}H_{64}O_{18}$	$[M+H]^+$	881.4165	881.4154	1.3	25	705.3819, 511.3410	22-β-Acetoxyglycyrrhizin	S
117	M89	57.3	$C_{42}H_{62}O_{17}$	$[M+H]^+$	839.4060	839.4044	1.6	25	663.3722, 469.3311	Licorice-saponin G2 or isomers	S
118	M89	57.7	$C_{42}H_{62}O_{17}$	$[M+H]^+$	839.4060	839.4054	0.6	15	663.3722, 487.3420, 469.3313	Licorice-saponin G2 or isomers	S

119	M72	58.5	$C_{15}H_{10}O_5$	$[M+H]^+$	271.0601	271.0604	-0.1	40	215.0712, 153.0188, 119.0496, 91.0548	Genistein or isomer	F
120	M92	58.6	$C_{12}H_{14}O_4$	$[M+H]^+$	223.0965	223.0969	-0.2	15	203.1803, 191.0708, 167.9943, 135.0812	Apiole	O
121	M89	58.8	$C_{42}H_{62}O_{17}$	$[M+H]^+$	839.4060	839.4044	1.6	25	663.3725, 487.3420, 469.3316, 451.3199	Licorice-saponin G2 or isomers	S
122	M89	59.4	$C_{42}H_{62}O_{17}$	$[M+H]^+$	839.4060	839.4034	1.5	25	663.3713, 645.3619, 627.3518, 487.3411, 469.3306, 451.3197	Licorice-saponin G2 or isomers	S
123	M93	59.9	$C_{42}H_{62}O_{16}$	$[M+H]^+$	823.4111	823.4090	0.3	25	647.3780, 471.3464, 453.3365	Glycyrrhizic acid	S
124	M94	60.8	$C_{42}H_{62}O_{16}$	$[M+H]^+$	823.4111	823.4097	0.2	20	647.3785, 453.3373	Uralsaponin A	S
125	M95	61.2	$C_{42}H_{62}O_{16}$	$[M+H]^+$	823.4111	823.4108	0.0	20	647.3798, 453.3365	Uralsaponin B	S
126	M96	66.3	$C_{27}H_{28}N_2O_4$	$[M+H]^+$	445.2122	445.2306	4.1	25	385.1897, 252.1021, 224.1076, 194.1184, 177.0917, 117.0707, 105.0341	Aurantiamide acetate	O

A [a], amino acids; N [n], nucleosides; PA [pa], phenolic acids; F [f], flavonoids; S [s], saponins; I [i], iridoids; O [o], other compounds

Table S2. The key unique pathways in ACP for the treatment of allergic diseases.

No.	Pathway	Function
1	PI3K-Akt signaling pathway	Regulates the proliferation of airway smooth muscle cells in asthma and is a potential pathway for the treatment of asthma [45].
2	Apoptosis	Regulated by T cells, it is involved in the occurrence and maintenance of allergic diseases [49].
3	Th17 cell differentiation	Th17 cells are one of the main cell lineages that cause allergic diseases, and the Treg/Th17 ratio is associated with allergic diseases [46].
4	TNF signaling pathway	TNF-α is associated with the occurrence of asthma [47].
5	Neurotrophin signaling pathway	It is associated with the onset of allergic asthma [50].
6	T cell receptor signaling pathway	Signal transduction changes/defects are associated with allergic diseases [48].
7	C-type lectin receptor signaling pathway	The interaction between C-type lectin receptors and allergens drives allergic reactions [51].
8	cAMP signaling pathway	cAMP regulates the balance between Th1/Th2 and relieves allergic diseases symptoms [52].
9	Focal adhesion	Not only does it influence the immediate degranulation of mast cells, but it also contributes to the modulation of immune cell behavior [53].
10	Ras signaling pathway	Participates in the activation of Th2 cells and is associated with peanut allergic diseases [54].

Table S3. The key common pathways in ACP for the treatment of allergic diseases.

No.	Pathway	Function
1	cGMP-PKG signaling pathway	Regulates contraction and relaxation of vascular smooth muscle in the airway wall [55].
2	Arachidonic acid metabolism	Plays an important role in allergic reactions [56].
3	Calcium signaling pathway	Potential therapeutic target for allergic asthma, regulates the function of Th1, Th2, and Th17 cells [57].
4	Salivary secretion	Children with allergic rhinitis have reduced saliva secretion [59].
5	Adrenergic signaling in cardiomyocytes	Key pathway for treating allergic diseases [58].

Table S4. Molecular docking results regarding the unique part (kcal/mol).

No. -unique	Mol ID	Degree	Compounds	MAPK1	MAPK3	PIK3CD	PIK3CB	AKT1	PIK3CA	PIK3R1	RELA	NFKB1	MAPK8
1	M12	7	Lyoniresinol	-7.2	-6.6	-6.4	-6	-6.1	-4.9	-6.3	-6.3	-5.1	-6.4
2	M54	5	Limonin	-6.4	-7.7	-8.6	-8.4	-8.4	-7	-8.4	-8.9	-7.7	-9
3	M26	5	7-Acetoxy-2-methylisoflavone	-9.3	-8.6	-8.1	-7	-7.3	-6.4	-8.1	-7.3	-6.3	-8.1
4	M92	4	Apiole	-6.4	-6.1	-5.6	-5.9	-5.1	-4.4	-6.3	-5.1	-5	-5.9
5	M88	4	Phellopterin	-7.8	-8.0	-7.7	-6.7	-6.7	-5.6	-7.4	-7.2	-6.1	-7.8
6	M87	4	Byakangelicol	-8.3	-7.9	-7.6	-6.7	-6.7	-5.5	-7.1	-7	-6	-7.5
7	M30	3	Caffeic acid	-6.5	-6.4	-6.1	-6.9	-6.9	-4.9	-6.8	-5	-5.6	-5.8
8	M64	2	Nomilin	-5.5	-7.0	-7.7	-7.8	-7.8	-5.7	-7.8	-8	-7.5	-8.5
9	M71	2	Naringenin	-8.6	-8.7	-7.9	-7.3	-7.3	-6.2	-8.7	-6.6	-6.9	-8.2
10	M45	2	Dillapiol	-6.3	-6.1	-6.0	-4.9	-6.6	-4.5	-6.2	-5.4	-4.7	-5.8
11	M86	2	Nortangeretin	-9.0	-8.8	-7.6	-7.3	-7.3	-6	-8.5	-7.1	-7.5	-8.1
12	M80	2	Luteolin	-8.9	-8.6	-8.0	-7.4	-7.4	-6.3	-8.8	-6.7	-6.9	-8.1
13	M81	2	Kaempferol	-9.1	-8.4	-7.5	-7.4	-7.4	-6.1	-7.9	-6.6	-6.8	-8
14	M85	2	Quercetin	-9.2	-8.9	-7.9	-7.4	-7.4	-6.3	-8.2	-6.5	-6.8	-8.1

Table S5. Molecular docking results regarding the common parts (kcal/mol).

No. -common	Mol ID	Degree	Compounds	ADRA1D	ADRB1	ADRA1A	ADRA2B	ADRA2A	HPGDS	ALOX5	TBXAS1	TBXA2R
1	M90	5	Naringin-3-*O*-(3-hydroxy-3-methylglutarate)-glucoside	-5.7	-7.4	-6.5	-6.1	-8.3	-7.9	-7.4	-7.1	-5.9
2	M93	4	Glycyrrhizic acid	-7.1	-6.5	-7.5	-7.6	-8.8	-8.3	-8.1	-11.6	-7.6
3	M20	4	Heptopyranosides	-6.3	-6.7	-7.3	-6	-6.4	-7.6	-6.8	-6.8	-6.7
4	M89	4	Licorice-saponin G2	-13.4	-15.7	-11.4	-12.6	-15.4	-12.5	-15.5	-13.0	-14.8
5	M94	3	Uralsaponin A	-13.6	-16.7	-12.0	-13.2	-16	-13.3	-15.9	-13.6	-14.3
6	M11	3	Phenylalanine	-6.0	-6.9	-6.4	-6.9	-6.2	-5.8	-6.6	-6.4	-5.9
7	M17	2	Tryptophan	-7.2	-7.5	-7.1	-7.1	-6.9	-7.0	-7.0	-6.8	-6.8
8	M73	2	Diosmin	-7.8	-9.3	-8.4	-8.8	-9.8	-9.0	-9.8	-9.4	-9.2
9	M50	2	Luteolin-7-*O*-*β*-D-glucoside	-8.8	-9.9	-8.2	-8.8	-9.7	-8.6	-9.3	-8.5	-9.3
10	M59	2	Hyperoside	-8.0	-7.8	-7.1	-7.8	-8.7	-7.6	-7.8	-7.6	-7.3
11	M55	2	Leucoside	-7.8	-8.6	-7.4	-7.3	-8.3	-8.5	-8.7	-8.3	-7.1
12	M65	2	Kaempferol 3-*O*-robinobioside	-9.0	-10.8	-7.2	-7.9	-9.6	-8.8	-7.8	-8.8	-7.0
13	M57	2	Rutin	-6.6	-10.9	-7.3	-8.2	-8.8	-8.7	-9.1	-8.5	-7.1
14	M72	2	Genistein	-7.9	-8.6	-6.8	-6.6	-8.3	-8.1	-7.9	-8.1	-8.2
15	M82	2	Daidzein	-7.4	-8.8	-7.1	-6.7	-8.7	-7.1	-8.0	-7.8	-8.2

References:

45. Liu, J.H.; Li, C.; Zhang, C.H.; Zhang, Z.H. LncRNA-CASC7 enhances corticosteroid sensitivity via inhibiting the PI3K/AKT signaling pathway by targeting miR-21 in severe asthma. *Pulmonology* **2020**, *26*, 18–26. https://doi.org/10.1016/j.pulmoe.2019.07.001.
46. Li, H.; Bradbury, J.A.; Dackor, R.T.; Edin, M.L.; Graves, J.P.; DeGraff, L.M.; Wang, P.M.; Bortner, C.D.; Maruoka, S.; Lih, F.B.; et al. Cyclooxygenase-2 regulates Th17 cell differentiation during allergic lung inflammation. *Am. J. Respir. Crit. Care Med.* **2011**, *184*, 37–49. https://doi.org/10.1164/rccm.201010-1637OC.
47. Bracke, M.; van de Graaf, E.; Lammers, J.W.J.; Coffer, P.J.; Koenderman, L. *In vivo* priming of Fc alpha R functioning on eosinophils of allergic asthmatics. *J. Leukoc. Biol.* **2000**, *68*, 655–661. https://doi.org/10.1189/jlb.68.5.655.
48. Chen, X.X.; Zhao, X.L.; Hu, Y.Z.; Zhang, B.W.; Zhang, Y.; Wang, S. Lactobacillus rhamnosus GG alleviates beta-conglycinin-induced allergy by regulating the T cell receptor signaling pathway. *Food Funct.* **2020**, *11*, 10554–10567. https://doi.org/10.1039/d0fo02124e.
49. Conejero, L.; Higaki, Y.; Baeza, M.L.; Fernández, M.; Varela-Nieto, I.; Zubeldia, J.M. Pollen-induced airway inflammation, hyper-responsiveness and apoptosis in a murine model of allergy. *Clin. Exp. Allergy* **2007**, *37*, 331-338. https://doi.org/10.1111/j.1365-2222.2007.02660.x.
50. Quinn, K.D.; Schedel, M.; Nkrumah-Elie, Y.; Joetham, A.; Armstrong, M.; Cruickshank-Quinn, C.; Reisdorph, R.; Gelfand, E.W.; Reisdorph, N. Dysregulation of metabolic pathways in a mouse model of allergic asthma. *Allergy* **2017**, *72*, 1327-1337. https://doi.org/10.1111/all.13144.
51. Chiffoleau, E. C-type lectin-like receptors as emerging orchestrators of sterile inflammation represent potential therapeutic targets. *Front. Immunol.* **2018**, *9*, 227. https://doi.org/10.3389/fimmu.2018.00227.
52. Jiang, T.Y.; He, F.; Han, S.W.; Chen, C.; Zhang, Y.N.; Che, H.L. Characterization of cAMP as an anti-allergic functional factor in Chinese jujube (*Ziziphus jujuba* Mill.). *J. Funct. Food.* **2019**, *60*, 103414. https://doi.org/10.1016/j.jff.2019.06.016.
53. Sutanto, H. Mechanobiology of Type 1 hypersensitivity: Elucidating the impacts of mechanical forces in allergic reactions. *Mechanobiol. Med.* **2024**, *2*, 100041. https://doi.org/10.1016/j.mbm.2024.100041.
54. Li, Y.X.; Kulis, M.; Pons, L.; Zhong, X.P.; Burks, A.W. Peanut allergen Ara h 2-specific T cells are activated via Ras-Erk MAP kinase pathway signalling and identified by CD154 expression. *Food Agric. Immunol.* **2011**, *22*, 335-344. https://doi.org/10.1080/09540105.2011.579591.
55. Ekstedt, S.; Georén, S.K.; Cardell, L.O. Effects of MP-AzeFlu enhanced by activation of bitter taste receptor TAS2R. *Allergy Asthma Clin. Immunol.* **2020**, *16*, 45. https://doi.org/10.1186/s13223-020-00438-w.
56. Hanna, V.S.; Hafez, E.A.A. Synopsis of arachidonic acid metabolism: A review. *J. Adv. Res.* **2018**, *11*, 23–32. https://doi.org/10.1016/j.jare.2018.03.005.
57. Pelletier, L.; Savignac, M. Calcium signaling components as potential therapeutic targets in allergic asthma. *Rev. Fr. Allergol.* **2013**, *53*, 129–132. https://doi.org/10.1016/j.reval.2013.01.009.
58. Morris, S.C.; Perkins, C.; Potter, C.; Parsons, D.; Schuman, R.; Khodoun, M.V.; Samavedam, U.; Strait, R.; Finkelman, F.D. Optimizing drug inhibition of IgE-mediated anaphylaxis in mice. *J. Allergy Clin. Immunol.* **2022**, *149*, 671–684. https://doi.org/10.1016/j.jaci.2021.06.022.
59. Elad, S.; Heisler, S.; Shalit, M. Saliva secretion in patients with allergic rhinitis. *Int. Arch. Allergy Immunol.* **2006**, *141*, 276-280. https://doi.org/10.1159/000095297.
